# Selective degradation of hyperphosphorylated tau by proteolysis-targeting chimeras ameliorates cognitive function in Alzheimer’s disease model mice

**DOI:** 10.3389/fphar.2024.1351792

**Published:** 2024-06-11

**Authors:** Dongping Yao, Ting Li, Lu Yu, Mingxing Hu, Ye He, Ruiming Zhang, Junjie Wu, Shuoyuan Li, Weihong Kuang, Xifei Yang, Gongping Liu, Yongmei Xie

**Affiliations:** ^1^ State Key Laboratory of Biotherapy and Cancer Center, West China Hospital, Sichuan University and Collaborative Innovation Center of Biotherapy, Chengdu, China; ^2^ Key Laboratory of Ministry of Education of China and Hubei Province for Neurological Disorders, Department of Pathophysiology, School of Basic Medicine, The Collaborative Innovation Center for Brain Science, Tongji Medical College, Huazhong University of Science and Technology, Wuhan, China; ^3^ Department of Psychiatry and National Clinical Research Center for Geriatrics, West China Hospital, Sichuan University, Chengdu, China; ^4^ Shenzhen Key Laboratory of Modern Toxicology, Shenzhen Medical Key Discipline of Health Toxicology (2020–2024), Shenzhen Center for Disease Control and Prevention, Shenzhen, China; ^5^ Co-Innovation Center of Neuroregeneration, Nantong University, Nantong, China

**Keywords:** Alzheimer’s disease, hyperphosphorylated tau, PROTACs, targeted protein degradation, ubiquitine proteasome system

## Abstract

Alzheimer’s disease (AD) is one of the most common chronic neurodegenerative diseases. Hyperphosphorylated tau plays an indispensable role in neuronal dysfunction and synaptic damage in AD. Proteolysis-targeting chimeras (PROTACs) are a novel type of chimeric molecule that can degrade target proteins by inducing their polyubiquitination. This approach has shown promise for reducing tau protein levels, which is a potential therapeutic target for AD. Compared with traditional drug therapies, the use of PROTACs to reduce tau levels may offer a more specific and efficient strategy for treating AD, with fewer side effects. In the present study, we designed and synthesized a series of small-molecule PROTACs to knock down tau protein. Of these, compound **C8** was able to lower both total and phosphorylated tau levels in HEK293 cells with stable expression of wild-type full-length human tau (termed HEK293-htau) and htau-overexpressed mice. Western blot findings indicated that **C8** degraded tau protein through the ubiquitin–proteasome system in a time-dependent manner. In htau-overexpressed mice, the results of both the novel object recognition and Morris water maze tests revealed that **C8** markedly improved cognitive function. Together, our findings suggest that the use of the small-molecule PROTAC **C8** to degrade phosphorylated tau may be a promising therapeutic strategy for AD.

## Graphical Abstract

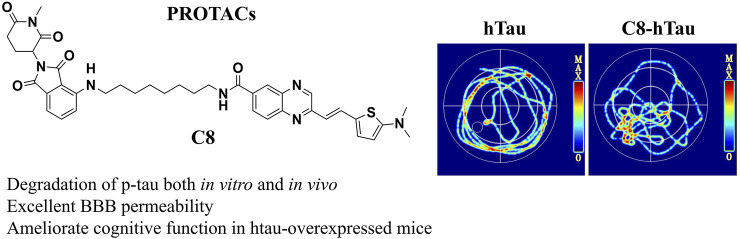

## 1 Introduction

Alzheimer’s disease (AD) is a degenerative brain disorder that progressively worsens over time (Grøntvedt et al., 2018). Its main symptoms include memory loss (Berntsen et al., 2022) and language difficulties (Robin et al., 2021), which are caused by the damage or destruction of nerve cells in specific parts of the brain. Since the disease was first reported by Alois Alzheimer in 1907, its incidence rate has been steadily increasing worldwide (Morgese et al., 2022), and the number of cases is estimated to exceed 150 million by 2050 (Vaz and Silvestre, 2020). However, the pathogenesis and mechanisms of AD are not yet fully understood (d’Errico and Meyer-Luehmann, 2020). Amyloid and tau proteins are considered the two primary neuropathological markers of AD (Lane et al., 2018). However, drugs directed at amyloid-β have failed to show clinical efficacy (Panza et al., 2018), leading researchers to focus more on the microtubule-associated protein tau (Congdon et al., 2018); the pathogenesis of AD is currently believed to be more closely correlated with tau pathology.

Tau is an essential protein in the neuronal cytoskeleton that is responsible for stabilizing microtubules (Oboudiyat et al., 2013). However, when hyperphosphorylated, the tau protein can form paired helical filaments (Xia et al., 2021), and further form neurofibrillary tangles (Gastard et al., 2003). This can result in microtubule dissociation in neurons, thus compromising axonal transport and diminishing synaptic function (Lane et al., 2018). Studies have demonstrated that phosphorylated tau (p-tau) has synergistic effects with amyloid protein in the pathogenesis of AD (Ittner and Götz, 2011). Current investigative tau-based therapeutic approaches have focused on inhibiting tau hyperphosphorylation (Basurto-Islas et al., 2014), reducing tau expression, targeting tau protein modifications (Chohan et al., 2006), inhibiting tau aggregation (Panza et al., 2016), clearing tau pathology, stabilizing microtubules (Zhang et al., 2012), promoting neuroregeneration to rescue neuronal function (Iqbal et al., 2018), and tau immunotherapies (Congdon et al., 2018). However, these approaches have potential limitations, and in many cases, the preclinical success of animal models has failed to translate into benefits for human patients. Recently, a new protein degradation technology for targeting and degrading toxic tau has emerged, in which small-molecule agents may represent a promising strategy for tauopathies (Bondeson et al., 2015; An and Fu, 2018; Xi et al., 2019; Li and Song, 2020; Li et al., 2022).

Proteolysis-targeting chimeras (PROTACs) are an emerging strategy for the degradation of proteins that are difficult to target with traditional small-molecule drugs (Liu et al., 2022). PROTACs comprise a ternary complex consisting of a linker connecting a ligand for the target protein and a ligand for E3 ligase (Yao et al., 2022). The PROTAC approach facilitates the binding of E3 ligase to the protein of interest, and then promotes the degradation of the protein of interest, which is ubiquitinated by the ubiquitin–proteasome system (Khan et al., 2020). Crews’ group designed the first small-molecule PROTACs to bind nutlin- 3a and induce its degradation via the mouse double minute 2 homolog (Schneekloth et al., 2008). They have also developed small-molecule PROTACs to induce the degradation of many disease-associated proteins (An and Fu, 2018). In 2018, Jiang et al. reported the first peptidic PROTACs that targeted tau proteins. These peptidic PROTACs were able to interact efficiently with tau proteins and induce their degradation (Lu et al., 2018). However, the use of peptidic PROTACs may limit their therapeutic potential because of their high molecular weight, unstable peptide linkage, poor cell penetration, low potency issues with instability, and poor blood–brain barrier (BBB) penetration (Chu et al., 2016; An and Fu, 2018). The development of small-molecule PROTACs for tau is therefore anticipated to mitigate the toxic effects of p-tau and safeguard neurons in AD (Kargbo, 2019; Silva et al., 2019; Pradeepkiran and Reddy, 2021; Wang et al., 2021; Wang et al., 2023; Zhang et al., 2023).

In the present study, we designed a series of compounds based on PROTAC principles. Of these compounds, **C8** significantly enhanced tau clearance via the ubiquitination–proteasome pathway in HEK293-htau cell models. Furthermore, **C8** markedly reduced both total tau and p-tau levels in htau-overexpressed mice, with an associated amelioration of cognitive function.

## 2 Materials and methods

### 2.1 Chemistry methods

All reagents were obtained from commercial suppliers and used without further purification. Reactions were carried out using magnetic stirrers and monitored for completion by thin-layer chromatography (Yantai Xinuo New Material Technology Co.) or Liquid Chromatograph Mass Spectrometer (Bruker Inc., Karlsruhe, Germany). Flash column chromatography (200–300 mesh) was used to purify the materials. A Bruker AV-400 (Bruker Inc., Karlsruhe, Germany) nuclear magnetic resonance (NMR) spectrometer was used to measure ^1^H and ^13^C NMR. All final compounds had a purity of at least 95% as determined by high-performance liquid chromatography (Waters Inc., Milford, MA, United States).

#### 2.1.1 Synthesis of compound **3**

Compound **1** (1.0 g, 1.0 eq) and compound **2** (1.0 g, 1.1 eq) were added to a vial containing glacial acetic acid (30 mL), and potassium acetate (1.8 g, 3.1 eq) was added while stirring. The reaction mixture was stirred for 16 h at 90°C. After cooling to room temperature, the mixture was filtered, and the filter cake was washed with water (30 mL × 3) and methanol (10 mL). The crude material was purified in a silica gel column (dichloromethane:methanol, 20:1) to yield compound **3** as a white solid (1.3 g, 81.2%).

#### 2.1.2 Synthesis of compound **5**

Compound **4** (3.3 g, 5 eq) was dissolved in anhydrous dichloromethane (20 mL), and (BOC)_2_O (1.0 g, 1.0 eq) was carefully added dropwise at 0°C. The reaction was then stirred at room temperature for 24 h. Upon reaction completion, the solvent was evaporated under reduced pressure. The crude product was dissolved in water (30 mL) and extracted with ethyl acetate (3 × 30 mL). Organic layers were combined and the product was purified by flash column chromatography (dichloromethane:methanol, 20:1) to yield compound **5** as a light-yellow solid (900 mg, 99%).

#### 2.1.3 Synthesis of compound **6**

Compound **3** (450 mg, 1.0 eq), and compound **5** (600 mg, 1.5 eq) were added to anhydrous *N,N*-dimethylformamide (10 mL), and triethylamine (0.6 mL, 3.0 eq) was added dropwise while stirring. The reaction mixture was stirred for 5 h at 90°C. Ethyl acetate (20 mL) was added and the organic layer was washed with brine (3 × 50 mL) and dried over MgSO_4_. The crude product was subjected to purification by silica gel (petroleum ether:ethyl acetate, 5:1) to yield compound **6** as a yellow-green solid (330 mg, 45%).

#### 2.1.4 Synthesis of compound **7**

Compound **6** (330 mg, 1.0 eq) was added to anhydrous dichloromethane (8 mL), and trifluoroacetic acid (2 mL, 20% in dichloromethane) was added dropwise while stirring. The reaction mixture was stirred for 2 h at room temperature. Upon completion of the reaction, the solvent was evaporated under reduced pressure. Ethyl acetate (20 mL) was added and the solution was washed with brine (3 × 50 mL) and dried over MgSO_4_. The crude product was subjected to purification by silica gel (dichloromethane:methanol, 20:1) to yield compound **7** as a yellow-green solid (190 mg, 76%).

#### 2.1.5 Synthesis of compound **10**

Compound **9** (1.57 g, 1.0 eq) was added to a solution of compound **8** (1.0 g, 1.0 eq) in glacial acetic acid (30 mL). The mixture was then heated at 110°C for 5 h. Next, the solvent was evaporated under reduced pressure, and the remaining reactants were dissolved in ethyl acetate (20 mL) and washed with saturated saline (20 mL × 3). The residue was purified by flash column chromatography (gradient of dichloromethane:methanol from 60:1 to 30:1) to yield compound **10** as a yellow liquid (4.0 g, 33%).

#### 2.1.6 Synthesis of compound **12**

Compound **10** (200 mg, 1.0 eq) and compound **11** (170 mg, 1.0 eq) were dissolved in a solution of 5 M sodium hydroxide (20 mL) containing Aliquat 336 (100 mg, 0.2 eq). The reaction mixture was heated at 110°C for 2 h. Next, the mixture was evaporated under reduced pressure, and the remaining reactants were dissolved in ethyl acetate (20 mL) and washed with saturated saline (20 mL × 3). The crude product was purified by flash column chromatography (gradient of dichloromethane:methanol from 60:1 to 20:1) to yield compound **12** as a pale-yellow solid (130 mg, 37%). ^1^H NMR (400 MHz, DMSO-*d*_6_) δ9.04 (s, 1H), 8.38 (d, *J* = 18.7 Hz, 1H), 8.21 (dd, *J* = 8.6, 1.6 Hz, 1H), 8.03 (dd, *J* = 15.6, 9.1 Hz, 1H), 7.89 (dd, *J* = 20.1, 8.6 Hz, 1H), 7.20 (dd, *J* = 9.4, 4.1 Hz, 1H), 6.67 (d, *J* = 15.5 Hz, 1H), 5.96 (t, *J* = 4.4 Hz, 1H), 3.02 (d, *J* = 2.8 Hz, 6H). Liquid chromatography electrospray ionization mass spectrometry (LC-ESI-MS), calcd. for C_38_H_41_N_7_O_5_S [M + H]^+^:326.1, found:326.1.

#### 2.1.7 Synthesis of compound **13**

*N*-hydroxysuccinimide (67 mg, 1.0 eq), 1-(3-dimethylaminopropyl)-3-ethylcarbodiimide hydrochloride (111 mg, 1.0 eq), and 4-dimethylaminopyridine (71 mg, 1.0 eq) were added to a stirred solution of compound **12** (200 mg, 1.0 eq) in dichloromethane (20 mL). The reaction mixture was stirred at room temperature for 24 h. After monitoring the complete reaction, the solvent was concentrated under reduced pressure before being used to synthesize compound **14**.

#### 2.1.8 Synthesis of compound **14**

Compound **7** (1.0 g, 1.0 eq) and compound **13** (1.0 g, 1.0 eq) were added to anhydrous dichloromethane (30 mL), and triethylamine (1.2 mL, 3.0 eq) was added dropwise. The reaction mixture was stirred for 24 h at room temperature. Upon completion of the reaction, the solvent was removed under reduced pressure. Ethyl acetate (20 mL) was added and the organic layer was washed with brine (3 × 50 mL) and dried over MgSO_4_. The residue was purified by column chromatography on silica gel (dichloromethane:methanol, 60:1) to yield compound **14** as a red solid (4.0 g, 48%).

**C2**: ^1^H NMR (400 MHz, CDCl_3_) δ 8.84 (s, 1H), 8.41 (s, 1H), 8.10 (t, *J* = 8.3 Hz, 1H), 7.92 (dd, *J* = 15.8, 8.5 Hz, 2H), 7.48 (dd, *J* = 17.7, 9.8 Hz, 2H), 7.15–6.95 (m, 4H), 6.64 (d, *J* = 15.4 Hz, 1H), 6.52 (d, *J* = 17.0 Hz, 1H), 4.94 (dd, *J* = 11.9, 5.0 Hz, 1H), 3.82–3.65 (m, 2H), 3.65–3.53 (m, 2H), 3.04 (d, *J* = 8.5 Hz, 6H), 2.29–1.92 (m, 4H). LC-ESI-MS, calcd. for C_38_H_41_N_7_O_5_S [M + H]^+^:624.2, found:624.2.

**C4**: ^1^H NMR (400 MHz, CDCl_3_) δ 8.84 (s, 1H), 8.43 (s, 1H), 8.13 (dd, *J* = 8.7, 1.6 Hz, 1H), 7.90 (dd, *J* = 14.7, 9.6 Hz, 2H), 7.46 (t, *J* = 7.8 Hz, 1H), 7.13–6.95 (m, 2H), 6.89 (d, *J* = 8.4 Hz, 1H), 6.78 (s, 1H), 6.64 (d, *J* = 15.4 Hz, 1H), 6.25 (t, *J* = 5.7 Hz, 1H), 5.80 (t, *J* = 5.6 Hz, 1H), 5.06–4.83 (m, 1H), 3.30 (s, 2H), 3.20 (q, *J* = 7.3 Hz, 2H), 3.03 (d, *J* = 5.8 Hz, 6H), 1.73 (s, 4H), 1.36 (dd, *J* = 13.5, 6.2 Hz, 2H), 0.87 (dd, *J* = 15.2, 7.9 Hz, 2H). ^13^C NMR (101 MHz, CDCl_3_) δ 171.63, 169.65, 167.62, 161.63, 152.95, 146.94, 145.65, 144.28, 136.19, 133.42, 132.50, 131.92, 128.84, 128.72, 127.16, 125.73, 117.06, 116.79, 111.72, 110.28, 102.62, 49.09, 47.42, 42.24, 39.67, 31.55, 29.70, 26.94, 26.34, 22.87, 8.74. LC-ESI-MS, calcd. for C_38_H_41_N_7_O_5_S [M + H]^+^:652.2, found:652.0.

**C6**: ^1^H NMR (400 MHz, CDCl_3_) δ 8.85 (s, 1H), 8.40 (d, *J* = 1.7 Hz, 1H), 8.13 (dd, *J* = 8.7, 1.9 Hz, 1H), 7.97–7.93 (m, 1H), 7.91 (d, *J* = 5.1 Hz, 1H), 7.54–7.40 (m, 1H), 7.12–7.00 (m, 2H), 6.88 (d, *J* = 8.5 Hz, 1H), 6.65 (d, *J* = 15.5 Hz, 1H), 6.57 (t, *J* = 5.6 Hz, 1H), 6.24 (t, *J* = 5.5 Hz, 1H), 5.81 (t, *J* = 3.7 Hz, 1H), 4.93 (dd, *J* = 12.2, 5.3 Hz, 1H), 3.72 (q, *J* = 7.0 Hz, 2H), 3.28 (dd, *J* = 12.6, 6.6 Hz, 2H), 3.04 (s, 6H), 2.95–2.62 (m, 4H), 1.66 (dd, *J* = 12.2, 6.1 Hz, 4H), 1.45 (dd, *J* = 9.0, 5.6 Hz, 4H). ^13^C NMR (101 MHz, CDCl_3_) δ 171.33, 169.58, 168.75, 167.67, 166.65, 161.61, 152.89, 147.03, 145.72, 144.25, 139.70, 136.13, 133.62, 133.38, 132.50, 131.88, 128.88, 128.72, 127.14, 125.73, 116.79, 111.45, 109.97, 102.61, 58.43, 48.99, 42.55, 42.25, 40.10, 31.50, 29.45, 28.93, 26.59, 26.50, 22.83, 18.43. LC-ESI-MS, calcd. for C_38_H_41_N_7_O_5_S [M + H]^+^:680.2, found:680.2.

**C8**: ^1^H NMR (400 MHz, CDCl_3_) δ 8.86 (s, 1H), 8.37 (d, *J* = 1.5 Hz, 1H), 8.14 (dd, *J* = 8.7, 1.7 Hz, 1H), 7.95 (dd, *J* = 12.1, 8.8 Hz, 2H), 7.56–7.37 (m, 1H), 7.06 (dd, *J* = 10.1, 5.6 Hz, 2H), 6.88 (d, *J* = 8.5 Hz, 1H), 6.66 (d, *J* = 15.4 Hz, 1H), 6.51 (t, *J* = 5.4 Hz, 1H), 6.25 (t, *J* = 5.4 Hz, 1H), 5.82 (d, *J* = 4.0 Hz, 1H), 4.92 (dd, *J* = 12.1, 5.3 Hz, 1H), 3.71 (dt, *J* = 20.6, 10.3 Hz, 1H), 3.59–3.37 (m, 6H), 3.37–3.10 (m, 3H), 1.36 (s, 12H), 0.88 (t, *J* = 6.0 Hz, 4H). ^13^C NMR (101 MHz, CDCl_3_) δ 171.21, 169.53, 168.65, 166.61, 161.60, 152.87, 147.05, 145.77, 144.24, 139.77, 136.10, 133.72, 133.36, 132.50, 131.86, 128.90, 128.70, 127.15, 125.73, 116.80, 116.73, 111.35, 109.88, 102.61, 53.43, 50.82, 48.94, 42.55, 42.25, 40.27, 38.61, 31.48, 29.69, 29.47, 28.90, 26.60, 22.82. LC-ESI-MS, calcd. for C_38_H_41_N_7_O_5_S [M + H]^+^:708.2, found:708.2.

**C10**: ^1^H NMR (400 MHz, CDCl_3_) δ 8.85 (s, 1H), 8.35 (d, *J* = 1.5 Hz, 1H), 8.11 (dd, *J* = 12.6, 11.2 Hz, 1H), 8.00–7.88 (m, 2H), 7.48 (t, *J* = 7.8 Hz, 1H), 7.05 (dd, *J* = 9.7, 5.6 Hz, 2H), 6.87 (d, *J* = 8.6 Hz, 1H), 6.65 (d, *J* = 15.5 Hz, 1H), 6.51 (d, *J* = 5.2 Hz, 1H), 6.22 (t, *J* = 5.3 Hz, 1H), 5.82 (d, *J* = 4.0 Hz, 1H), 5.03–4.81 (m, 1H), 3.79–3.54 (m, 1H), 3.54–3.36 (m, 4H), 3.35–3.11 (m, 3H), 3.04 (s, 6H), 2.37–2.02 (m, 2H), 1.88–1.52 (m, 10H), 0.87 (dd, *J* = 16.1, 9.1 Hz, 4H). ^13^C NMR (101 MHz, CDCl_3_) δ 169.50, 167.67, 166.62, 161.59, 152.83, 147.05, 145.77, 144.21, 139.79, 136.09, 133.77, 133.35, 132.49, 131.84, 128.89, 128.66, 127.18, 125.72, 116.79, 116.66, 111.29, 109.80, 102.60, 53.43, 48.91, 42.60, 42.24, 40.29, 38.61, 31.59, 31.47, 29.54, 29.25, 29.15, 29.10, 29.07, 28.99, 26.93, 26.76, 22.83. LC-ESI-MS, calcd. for C_38_H_41_N_7_O_5_S [M + H]^+^:736.2, found:736.2.

### 2.2 Antibodies and chemicals

The following antibodies were used in the present study: anti-pS262 (Signalway Antibody, 11111), anti-pS396 (Signalway Antibody, 11102), anti-pS404 (Signalway Antibody, 11112), T22 (anti-tau, Millipore, ABN454), AT8 (Thermo Fisher Scientific, MN1020), Tau5 (Abcam, ab80579), and DM1A (ABclonal, A7277). MG132 (MedChemExpress, HY-13259), okadaic acid (OA; Millipore, 19-130), and bafilomycin A1 (Abcam, ab120497) were dissolved in 99.7% DMSO (Sigma-Aldrich, D2650). We also used cycloheximide (CHX; MedChemExpress, HY-12320).

### 2.3 Cell cultures and treatments

HEK293 cells with stable expression of wild-type full-length human tau (termed HEK293-htau)were cultured in Dulbecco’s Modified Eagle Medium (DMEM) (Gibco, 11995500) containing 10% fetal bovine serum (Gibco, 2327864) and 200 mg/mL G418 (Thermo Fisher Scientific, 10131035) in a humidified incubator at 37 °C with a 5% CO_2_-containing atmosphere. The cells were placed into 12-well plates and cultured with DMEM containing the compounds at different concentrations with or without 5 μM MG132 or 100 nM bafilomycin A1 for 24 h at 37°C. To promote tau aggregation, cells were then cultured with DMEM containing 30 nM OA. To inhibit protein synthesis, cells were cultured with DMEM medium containing 100 μg/mL CHX with or without 0.05 μM **C8**. SH-SY5Y human neuroblastoma cells were cultured in 90% DMEM/F-12 (HyClone, SH30023.01) with 10% fetal bovine serum and penicillin (100 U/mL)/streptomycin (100 μg/mL) in a 37°C incubator with a humidified atmosphere of 5% CO_2_.

### 2.4 Cell viability assay

Cell viability was assessed using a Cell Counting Kit 8 (CCK-8) according to the manufacturer’s instructions. The cells were inoculated in 96-well plates at a concentration of 5,000 cells/well before being treated with various concentrations of compounds (0, 0.001, 0.01, 0.1, 1, and 10 μM) for 24 h. After the treatments, the medium was discarded and 10 μL of CCK-8 was added to 90 μL of the medium. After incubation for 30 min at 37°C, absorbance was measured at 450 nm using a microplate reader (BioTek, 250058). At least three independent experiments were conducted.

### 2.5 *In vivo* imaging

Normal BALB/C nude mice (*n* = 3, male, 6 weeks) were anesthetized with isoflurane and intravenously injected with **C8** (3.0 mg/kg, in 45% DMSO and 55% propylene glycol) in the tail. Images were recorded at 0, 10, 20, 30, and 60 min after injection using an IVIS Lumina Series III imaging system (PerkinElmer, Waltham, MA, United States) with excitation at 534 nm and emission at 716 nm.

### 2.6 Animals and drug administration

Male C57BL/6 mice (8 weeks old, 23 ± 2 g) were purchased from Experimental Animal Central (Beijing Vital River Laboratory Animal Technology Co., Ltd.) and acclimated for 1 week. Mice were then anesthetized with isoflurane and placed in a stereotaxic apparatus for the bilateral injection of purified pAAV-hSyn-htau-mCHERRY-3×FLAG (constructed and synthesized by Shanghai He Yuan Co.) virus (1.0 μL) into the hippocampal CA1 region (−2.2 mm posterior, ±2.5 mm lateral, −2.4 mm ventral). One month after receiving the htau virus injection, mice were administered **C8**. The **C8** was dissolved in 5% DMSO +50% polyethylene glycol 400 + 45% physiological saline, and was administered intraperitoneally to reach a final concentration of 5 or 10 mg/kg. Mice were dosed three times a week for 1 month. All mice were maintained at a temperature of 24°C ± 2°C with free access to food and water under a 12 h light/dark cycle. At the end of the C8 treatment, the 10 mg/kg dose group was selected to undergo behavioral evaluations. In addition, the hippocampi were separated from the brains of mice in both the 5 and 10 mg/kg dose groups, and were used to detect tau clearance. All animal experiments were conducted according to the Guidelines for the Care and Use of Laboratory Animals of the Ministry of Science and Technology of the People’s Republic of China and approved by the Institutional Animal Care and Use Committee at Tongji Medical College, Huazhong University of Science and Technology.

### 2.7 Novel object recognition (NOR) test

The NOR test is a learning/memory test that is based on the principle that animals have an innate tendency to explore new objects. The experiment was conducted in a 50 cm × 50 cm square box with three objects (A, B, and C); object A was the same as object B, whereas object C was different. On day 1, each mouse was habituated to the chamber, without any objects, for 5 min. On day 2, each mouse was placed at the same starting point, re-entered the opaque box containing two objects (A and B), and was allowed to explore for 5 min. On day 3, object B was replaced with novel object C in the same position, and each mouse was allowed to explore both objects for 5 min. The chamber and objects were thoroughly cleaned with 75% ethanol between trials. The recognition index was calculated as the time spent exploring the novel object divided by the total time spent exploring both objects. Exploration was defined as sniffing or touching the object with the nose and/or forepaws.

### 2.8 Morris water maze (MWM) test

The MWM test is the most commonly used behavioral task for assessing spatial learning and memory. The test was conducted in a round water tank that was divided into four quadrants, with a submerged escape platform (10 cm × 10 cm) placed in one quadrant (referred to as the target quadrant). The platform was positioned 1.5 cm below the surface of the opaque water, which was maintained at 22°C–25°C. Each mouse was trained to find the hidden platform for 6 consecutive days, with three trials per mouse per day between 3:00 p.m. and 8:00 p.m. In each training trial, the mouse started from one of four quadrants (excluding the target quadrant), and the trial was completed as soon as the mouse climbed onto the platform or when 60 s had elapsed. If the mouse failed to find the submerged escape platform within 60 s, the mouse was gently guided to the submerged platform and allowed to remain there for 30 s. The latency to find the platform was recorded, with the maximum escape latency recorded as 60 s (when the platform was not found). During the detection period, spatial memory was tested by removing the submerged platform and placing the mice in a random quadrant. The latency to enter the platform area for the first time, the time spent in the target quadrant, and the number of platform crossings were recorded.

### 2.9 Western blotting

For Western blot assays, cells were used or the hippocampi were separated from brains of mice. Cells or tissue were first homogenized and lysed at 0°C for 30 min in Western blot buffer (Beyotime, P0013) supplemented with protease inhibitor and phosphatase inhibitor cocktails. This was followed by denaturing, by boiling and sonication in a water bath, and centrifugation at 20,000 ×*g* for 20 min. Supernatants were then collected and protein concentrations were analyzed using a Pierce Bicinchoninic Acid Protein Assay Kit (Thermo Fisher Scientific, 23227). Proteins were separated by 10% sodium dodecyl sulfate-polyacrylamide gel electrophoresis and transferred to nitrocellulose membranes for 1 h. Next, membranes were blocked for 1 h with 5% (weight/volume) bovine serum albumin (BSA) dissolved in PBS, incubated overnight with primary antibody at 4°C, and then incubated with IRDyeTM (800 CW) conjugated with anti-mouse (800 M) or anti-rabbit IgG (800 R) for 1 h at room temperature. The immunoreactive bands were visualized and analyzed using an odyssey infrared imaging system.

### 2.10 Immunohistochemistry and immunofluorescence

Paraffin-embedded tissue samples were serially sectioned at 5 μm, heated at 65°C for 60 min, dewaxed in xylene, and rehydrated through graded ethanols (100%, 95%, 90%, 80%, and 75%). Sections were then treated with sodium citrate antigen repair solution (pH = 6.0) for 20 min at 95°C before being washed three times for 5 min each with 0.1% Triton X-100 PBS. Sections were treated with 3% hydrogen peroxide for 20 min at room temperature to block endogenous peroxidases, and then underwent three 5 min washes with 0.1% Triton X-100 PBS. Next, 0.5% Triton X-100 was used at room temperature for 40 min to break the membranes, and sections were blocked in 5% BSA for 40 min. The sections were then incubated overnight in primary antibody before being washed three times for 5 min each with 0.1% Triton X-100 PBS.

For samples that were used for immunohistochemistry, the sections were then incubated with corresponding horseradish peroxidase-conjugated secondary antibodies at room temperature for 1 h. After three 5 min washes with 0.1% Triton X-100 PBS, 3,3′-diaminobenzidine staining was performed for 1–2 min (under microscopic observation), and sections were again washed three times with 0.1% Triton X-100 PBS. Sections were then dehydrated through graded ethanols (75%, 80%, 90%, 95%, and 100%), cleared in xylene for 30 min, and sealed with neutral gum.

For sections that were used for immunofluorescence, the sections were incubated with the corresponding fluorescent secondary antibody at room temperature for 1 h before being washed three times for 5 min each with 0.1% Triton X-100 PBS. Next, cell nuclei were stained with 4′,6-diamidino-2-phenylindole for 10 min, and sections were washed three times. Finally, sections were blocked with a glycerol/PBS solution (1:1).

### 2.11 Statistical analysis

All data were collected and analyzed in a blinded manner. The data are presented as the mean ± standard error of the mean (SEM) or standard deviation. Statistical analyses of differences between groups were performed using two-way analysis of variance or Student’s t-test; these were followed by Tukey’s multiple comparisons test for multiple comparisons among more than two groups. Data were analyzed using GraphPad Prism software. *p* < 0.05 was considered significant.

## 3 Results and discussion

### 3.1 Design and synthesis of tau PROTAC degraders

**FIGURE 1:**
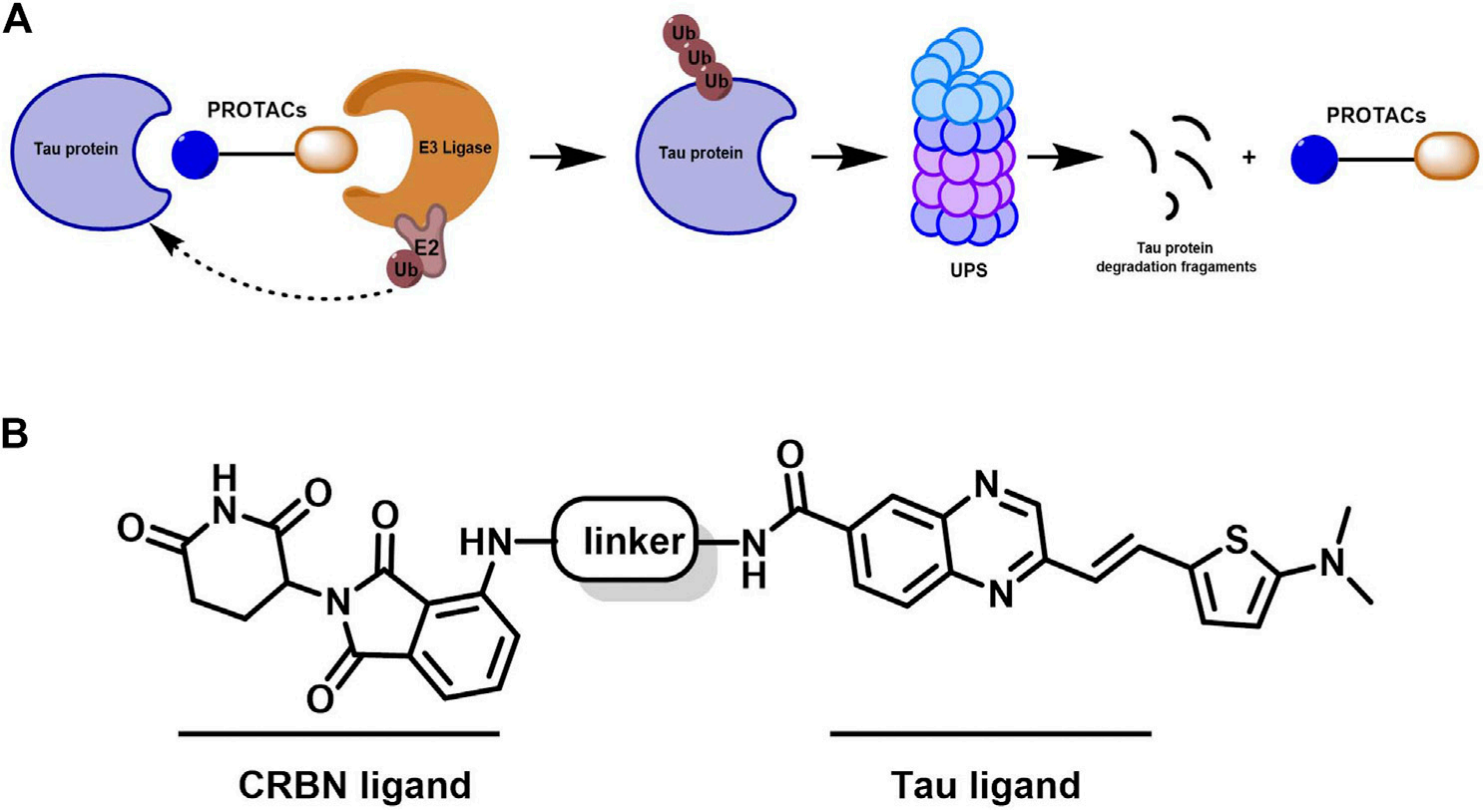
Mechanism of PROTAC technology and design for a new tau-targeting PROTAC degrader. **(A)** Working model of PROTACs. **(B)** PROTACs were synthesized to contain quinoxaline with a thiophene ring as a core scaffold for tau recognition, thalidomide for E3 ligase engagement, and an optimized hydrophobic alkyl linker for maximum target degradation efficiency.

**SCHEME 1:**
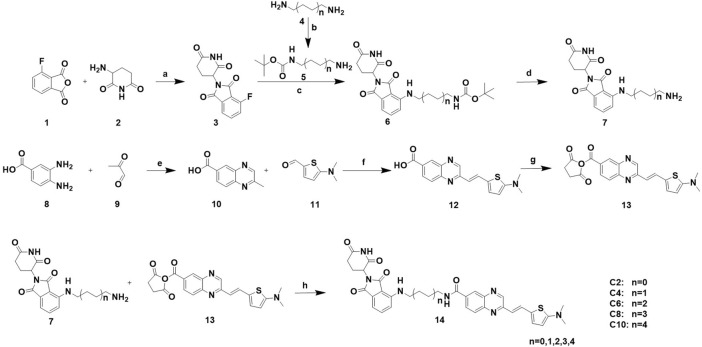
Synthesis of PROTACs. Reaction conditions: a: AcOK, AcOH, 90°C, 16 h b: (BOC)_2_O, rt, 24 h. c: Et_3_N, DMF, 90°C, 5 h. d: 20% CF_3_COOH, DCM, rt, 2 h. e: AcOH, relux, 5 h. f: Aliquat 336, 5 M NaOH, 110°C, 2 h. g: NHS, EDCI, DMAP, DCM, rt, 24 h. h: Et_3_N, DCM, rt, 24 h.

To achieve selective and efficient proteolysis of tau, we designed and synthesized hetero-bifunctional molecules containing a linker, coupled to a tau binder and E3 ligand, to facilitate the binding of E3 ligase to tau proteins. Upon tagging the tau proteins with ubiquitin, they were degraded by the proteasome (Figure 1A). Fluorescence probes of quinoxaline with a thiophene ring attached as a tau binder have been previously reported to most effectively detect tau aggregates in the brain, both *in vitro* and *in vivo*. Furthermore, substitution of the phenyl group of quinoxaline with other functional groups does not significantly affect the ability to bind tau (Zhu et al., 2018). Thalidomide was used as the E3 ligase binder in the present study. Because BBB permeability is important for AD treatment, we chose a long-chain alkyl group with hydrophobic properties as the linker of the tau binder and E3 ligand (Figure 1B). Intermediate compounds **7** and **12** were used to synthesize the target compound **14**. The synthetic route of tau PROTAC development is shown in Scheme 1. The structures were characterized using ^1^H NMR, ^13^C NMR, and LC-ESI-MS. The spectra are provided in the Supplementary Figures S1–S16.

### 3.2 Tau PROTAC inhibitors reduce p-tau levels in HEK293-htau cells

The CCK-8 assay was used to analyze the cytotoxicity of compounds **C2-C10** and the negative control compound **12** in HEK293-htau cells for 24 h (Supplementary Figure S17)). **C2**, **C6**, and **C8** slightly affected cell cytotoxicity even at concentrations as high as 10 μM. **C2**, **C10**, and **12** exhibited different levels of cytotoxicity at concentrations above 1 µM. No compounds showed cytotoxicity at 0.1 µM in the cytotoxicity test. Therefore, we used a concentration of 0.1 µM for subsequent experiments.

**FIGURE 2:**
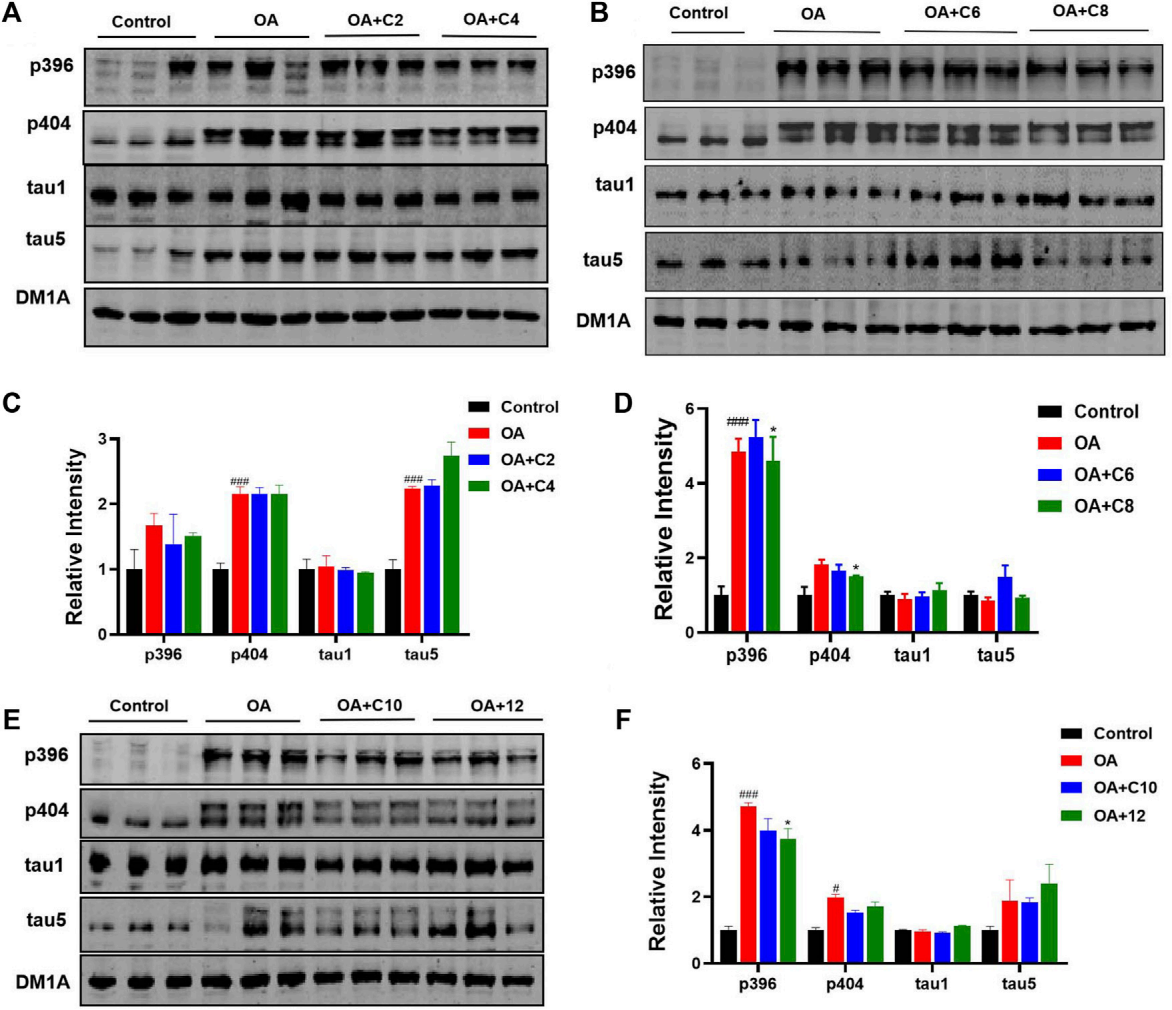
P-tau and total tau inhibitory activity of PROTACs. HEK293-htau cells were treated with OA (30 nM), OA (30 nM) + PROTACs (**C2**, **C4**, **C6**, **C8**, and **C10**), or OA (30 nM) + **12** (negative control compound) for 24 h. The levels of p-tau and total tau were then determined using Western blot analysis. **(A, C)** The degradation and resulting statistics of tau proteins by **C2** and **C4**. **(B, D)**The degradation and resulting statistics of tau proteins by **C6** and **C8**. **(E, F)** The degradation and resulting statistics of tau proteins by **C10** and **12**. Values represent the mean ± SEM; *p < 0.05, **p < 0.01, ***p < 0.001 vs. OA; ^#^ *p* < 0.05, ^##^ *p* < 0.01, ^###^ *p* < 0.001 vs. control. *n* = 3.

Next, we measured the effects of tau PROTACs on tau clearance in HEK293-htau cells. OA is a selective and potent inhibitor of the serine/threonine (Ser/Thr) phosphatases 1 and 2A (Cohen et al., 1990); it is extracted from the sponge *Hallichondria okadaii*. It induces the hyperphosphorylation of tau proteins and leads to pathological neuronal cell death, similar to that of AD (Pan et al., 2021). To evaluate the extent of PROTAC-triggered tau protein degradation, HEK293-htau cells were pretreated with OA (30 nM) and incubated with PROTACs at a concentration of 0.1 µM for 24 h before total tau and p-tau levels were detected using Western blot assays (Figure 2). **C2** and **C6** did not induce the degradation of p-tau or total tau protein. However, **C4** decreased the levels of tau phosphorylation at Ser396 sites, with no significant degradation of the other p-tau proteins or total tau. Moreover, **C8** reduced p-tau-Ser396 and p-tau-Ser404. By contrast, **C10** and **12** did not affect tau phosphorylation or total tau levels. Together, our findings indicate that **C4** and **C8** are the most effective for degrading p-tau in HEK293-htau cells. Furthermore, the chain length of PROTACs had a substantial impact on the degree of tau degradation.

**FIGURE 3:**
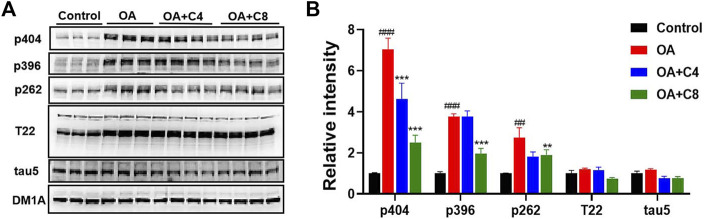
P-tau and total tau inhibitory activity of C4 and C8. **(A)** HEK293-htau cells were treated with OA (30 nM) and OA (30 nM) + **C4** or OA (30 nM) + **C8** for 24 h. The levels of p-tau and tau5 were then determined using Western blot analysis. **(B)** The resulting statistics of tau proteins by **C4** and **C8**. Values represent the mean ± SEM; *p < 0.05, **p < 0.01, ***p < 0.001 vs. OA; ^#^ *p* < 0.05, ^##^ *p* < 0.01, ^###^ *p* < 0.001 vs. control. *n* = 3.

Next, we measured the effects of **C4** and **C8** on tau clearance in HEK293-htau cells (Figure 3). **C4** reduced p-tau-Ser404, p-tau-Ser262, and total tau levels by 34.3%, 33.3%, and 36.4%, respectively. **C8** reduced p-tau-Ser404, p-tau-Ser396, and p-tau-Ser262 levels by 64.2%, 47.6%, and 33.3%, respectively. Furthermore, **C8** reduced tau (T22 antibody) and total tau5 levels by 41.6% and 34.4%, respectively. These results suggest that **C8** is significantly better than **C4** at reducing p-tau and total tau protein levels.

### 3.3 Optimal concentration of C8 for degrading of tau protein

**FIGURE 4:**
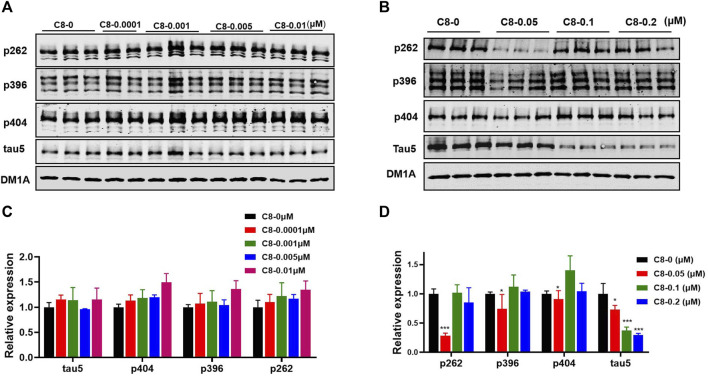
At multiple doses, C8 induced total tau and p-tau clearance in HEK293-htau cells. **(A, B)** C8 induced total tau and p-tau clearance as observed using Western blotting. **(C, D)** The resulting statistics of tau proteins by **C8**. Values represent the mean ± SEM; *p < 0.05, **p < 0.01, ***p < 0.001. *n* = 3.

To determine the optimal concentration of **C8**, we treated HEK293-htau cells with increasing concentrations of **C8** for 24 h before analyzing the lysates using Western blot assay (Figure 4). To screen for compounds that rapidly lowered tau, OA (30 nM) pretreatment was used to increase p-tau in HEK293-htau cells before the compound was administered. After screening for the tau-lowering effects of **C8**, HEK293-htau cells (without OA treatment) were used for further validation, and no OA pretreatment was added in the subsequent experiments. At a concentration of 0.05 µM, **C8** effectively decreased p-tau levels at Ser262 and Ser396 sites by 72.0% and 25.4%, respectively. At concentrations of 0.1 µM and 0.2 µM, **C8** was able to decrease tau5 levels by 62.5% and 70.4%, respectively. These findings indicate that **C8** can effectively decrease p-tau and total tau protein levels in HEK293-htau cells. Furthermore, we obtained similar experimental results in SH-SY5Y cells; **C8** degraded OA-induced p-tau levels, and optimal p-tau protein degradation was observed at a concentration of 0.05 µM (Supplementary Figure S18).

### 3.4 C8 induces tau clearance in a time-dependent manner via the ubiquitin-proteasome system

**FIGURE 5:**
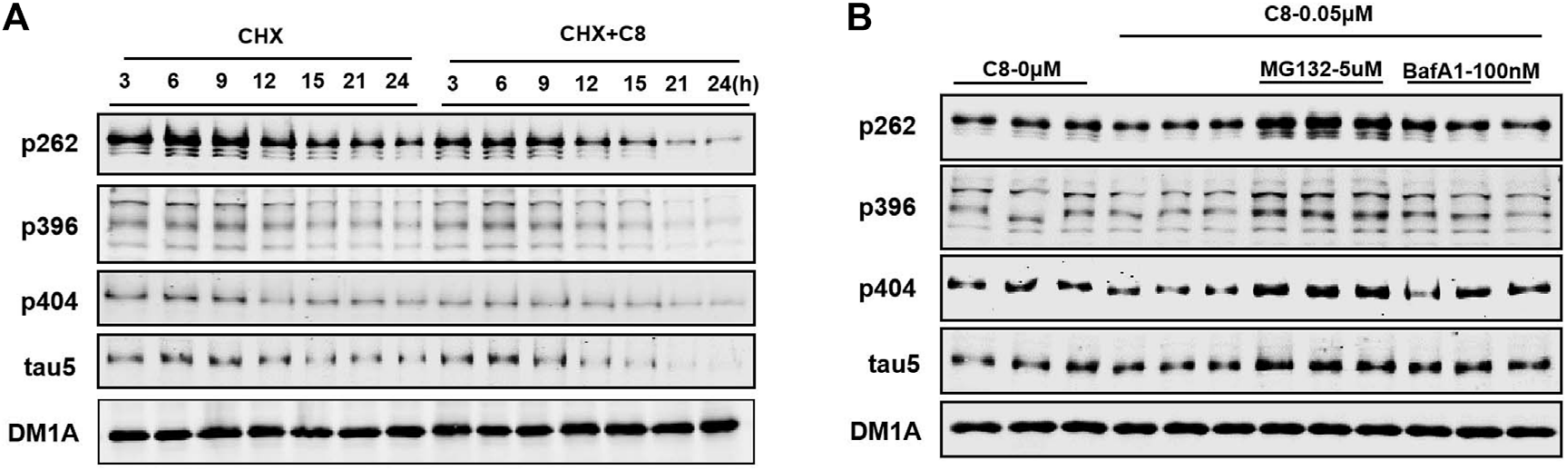
C8 induced tau clearance in a time-dependent manner via the ubiquitin-proteasome system. **(A)** C8 (0.05 µM) for 24 h induced tau reduction in HEK293-htau cells, as measured by Western blot. **(B)** The mechanism of **C8** induced tau clearance was dependent on the ubiquitin–proteasome system. HEK293-htau cells were pre-treated for 2 h with the proteasome inhibitor MG132 and the autophagy inhibitor bafilomycin A1; this was followed by 24 h of treatment with **C8** (0.05 µM). Total tau and p-tau levels were evaluated using Western blotting. *n* = 3.

Next, we conducted time-series studies to assess the degradation of tau proteins (Figure 5A). Cycloheximide (CHX) was first discovered in the bacterium *Streptomyces griseus*, and has activity against various eukaryotic algae and fungi (Syuhada et al., 2022). HEK293-htau cells were treated with both CHX and **C8** for 24 h, and tau protein expression was then detected using Western blot assay. After 12 h of treatment with **C8** at a concentration of 0.05 µM, a substantial amount (>50%) of p-tau degradation was achieved. Maximum p-tau degradation was observed after 24 h of **C8** treatment. Additionally, there was a significant decrease in total tau levels after 24 h of treatment. These findings imply that **C8** can reduce p-tau and total tau levels in a time-dependent manner, thus indicating that **C8** is an effective tau degrader.

To further determine the degradation pathway of **C8**, HEK293-htau cells were exposed to the proteasome inhibitor MG132 and the autophagy-lysosome inhibitor bafilomycin A1. **C8-**induced tau clearance in HEK293-htau cells was completely abolished by treatment with MG132, whereas bafilomycin A1 treatment did not affect tau clearance at all (Figure 5B). These findings suggest that **C8** can robustly reduce tau proteins via proteasomal degradation, but that this process is independent of the autophagic pathway, at least *in vitro*.

### 3.5 *In vivo* evaluation of the BBB permeability of compound C8

**FIGURE 6:**
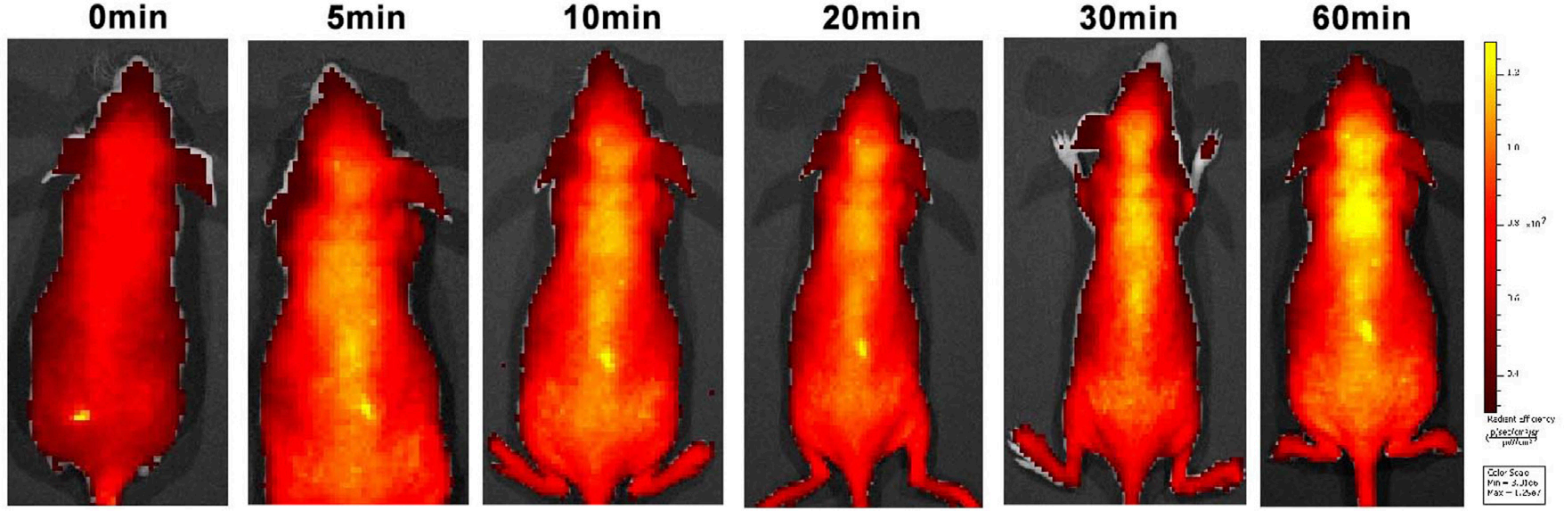
Small animal fluorescence imaging. Normal BALB/C nude mice (*n* = 3, male, 6 weeks old) were intravenously injected with **C8** (3.0 mg/kg). A semiquantitative analysis of fluorescence intensity in the brain region was conducted (excitation = 534 nm; emission = 716 nm).

Good BBB permeability helps to increase drug concentrations in the central nervous system. An *in vivo* imaging study was therefore conducted to investigate the BBB permeability of C8 (Figure 6). *In vivo* imaging in mice showed a gradual increase in fluorescence at 10 min, with fluorescence in the brain peaking at 60 min. These findings indicate that **C8** has excellent BBB permeability.

### 3.6 C8 improves cognition in htau-overexpressed mice

**FIGURE 7:**
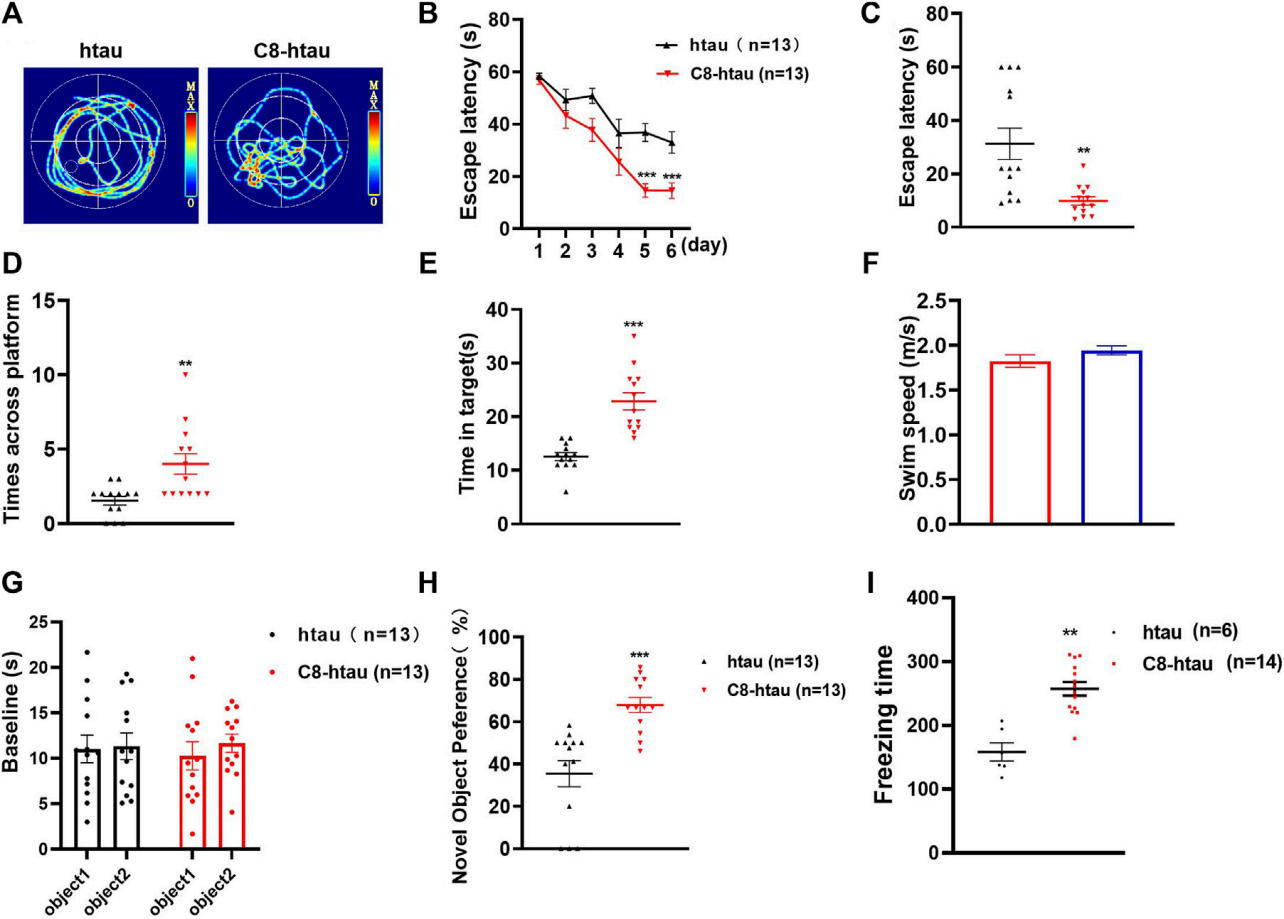
C8 improved cognitive function in htau-overexpressed mice. **(A–C)** In the MWM test, **C8** reduced the latency to find the platform during the learning trial. **(D–F)** The **C8** treated mice had increased platform crossings, target zone crossings and swimming speeds during the probe trial. **(G–I)** In the NOR test, **C8** markedly attenuated cognitive deficits. Values represent the mean ± SEM; *p < 0.05, **p < 0.01, ***p < 0.001 (*n* = 6–13 for each group).

To investigate the effects of **C8** on learning and memory, the MWM (Ennaceur and Delacour, 1988) and NOR tests (Morris, 1981) were used in htau-overexpressed mice. In the MWM test, memory is evaluated based on the search for survival in a pool, and spatial orientation is assessed based on the escape from the water; the NOR test assesses learning through the curiosity of animals for new stimuli (Lissner et al., 2021). Previous studies have reported that 12-month-oldhtau-overexpressed mice have memory deficits (Ma et al., 2013). We therefore stereotactically injected viruses overexpressing htau into the brains of mice (Wegmann et al., 2019). Next, **C8** (10 mg/kg) was intraperitoneally injected into the htau-overexpressed mice, and memory and learning were assessed using the MWM and NOR tests. In the MWM test, the latency to find the platform during the 6-day learning trial was significantly shortened by **C8** treatment (Figures 7A–C). Moreover, the **C8-**treated htau-overexpressed mice had increased platform crossings, target zone crossings, and swimming speeds during the probe trial (Figures 7D–F). We also observed that **C8** markedly attenuated cognitive deficits in the htau-overexpressed mice, as evidenced by the increased time spent exploring new objects in the NOR test (Figures 7G–I). Together, these data indicate that **C8** is effective for ameliorating cognitive function in the htau-overexpressed mice.

### 3.7 C8 promotes tau clearance *in vivo*

**FIGURE 8:**
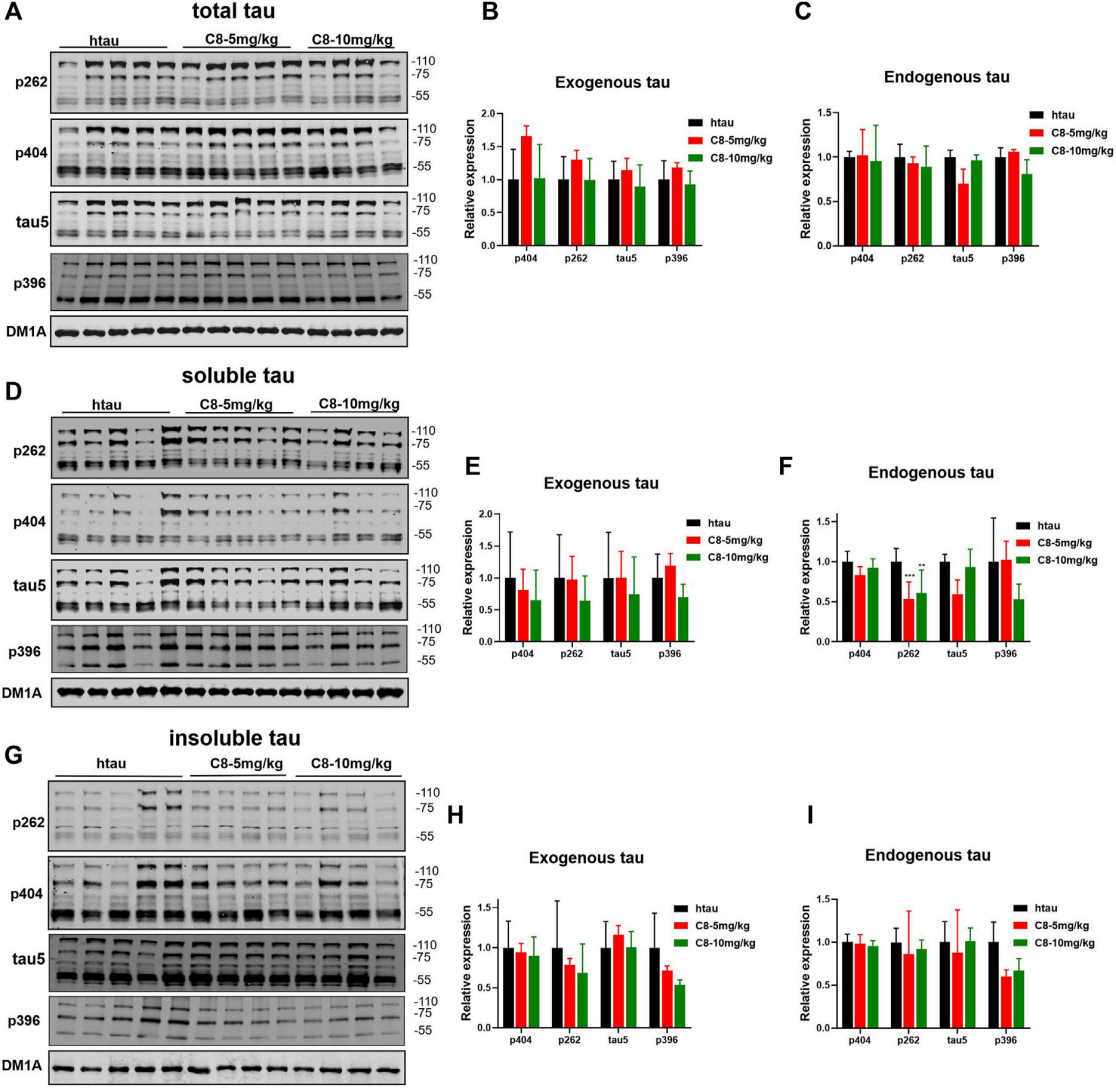
Intraperitoneally injected C8 reduced hippocampal tau in htau-overexpressed mice. C8 was administrated to htau-overexpressed mice through intraperitoneal injection for 1 month before hippocampal extracts were prepared for Western blotting. **(A–C)** Total tau was degraded (Western blot and statistical analyses). **(D–F)** Soluble tau was degraded (Western blot and statistical analyses). **(G–I)** Insoluble tau was degraded (Western blot and statistical analyses). 110 KD for exogenous tau and 55 KD for endogenous tau. Values represent the mean ± SEM; *p < 0.05, **p < 0.01, ***p < 0.001. *n* = 4**–**5.

**FIGURE 9:**
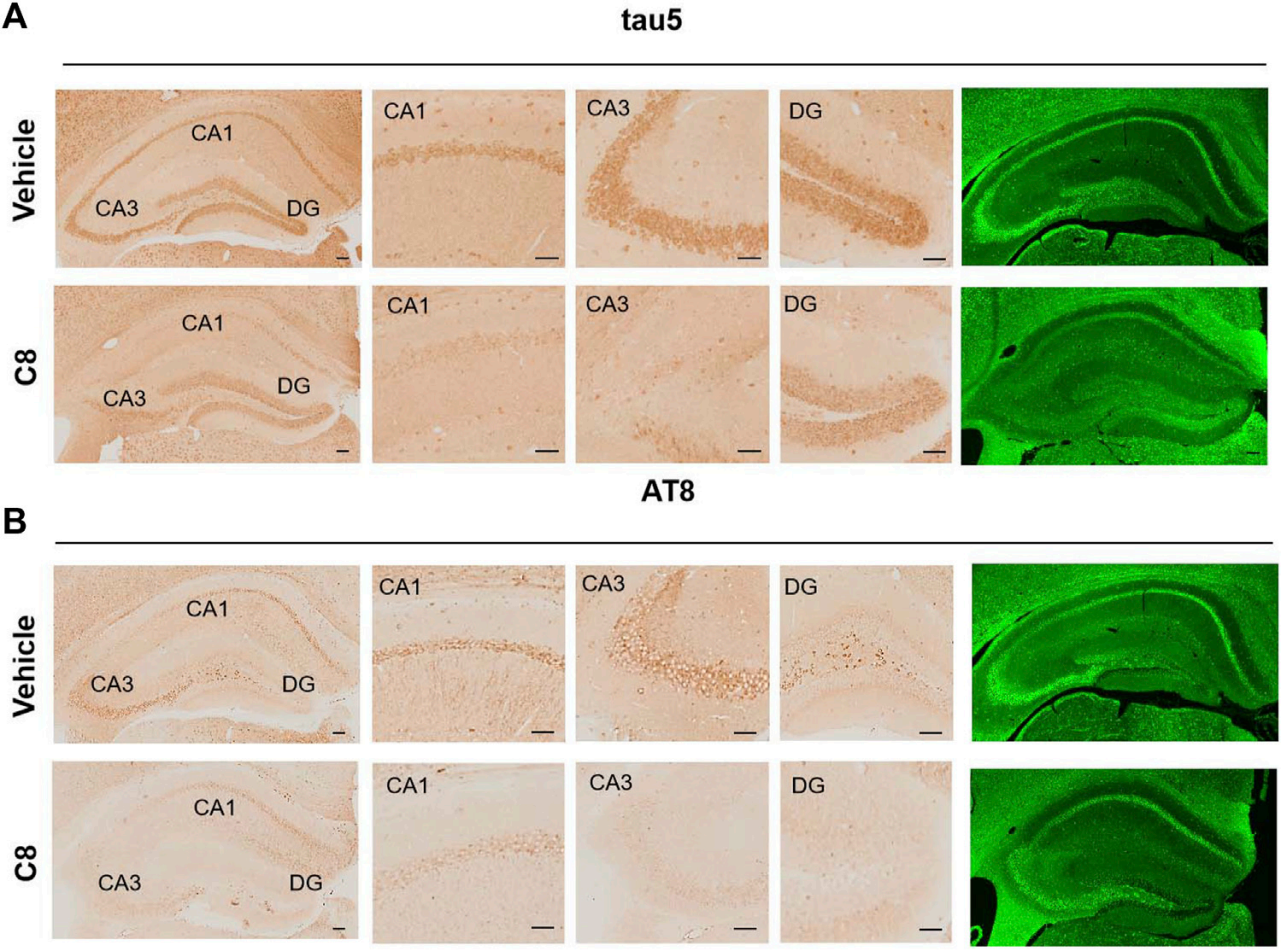
**C8** decreased tau in the hippocampal CA1, CA3, and DG regions, as measured by immunohistochemistry and immunofluorescence. Scale bar: 50 μm. *n* = 4 brain slices/3 mice.

To explore whether **C8** can also promote tau clearance *in vivo*, tau protein levels were detected in hippocampal tissue using Western blotting. Compared with the vehicle control, **C8** significantly reduced total tau and p-tau levels in the hippocampal extracts (Figures 8A–C). This result further indicated that **C8** can induce a sustained reduction of tau. Using Western blotting analyses, we observed that the intraperitoneal delivery of **C8** reduced both soluble and insoluble tau, although it was more efficient for tau in the soluble fraction (Figures 8D–I). A reduction of p-tau and total tau aggregation in the **C8**-treated group was also shown using tau5 and tau (AT8 antibody) immunofluorescence (Figure 9).

## 4 Conclusion

We successfully applied PROTAC technology to the targeted degradation of total tau and p-tau both *in vitro* and *in vivo*. With appropriate modifications of quinoxaline-based fluorescence probes with a thiophene moiety, five novel tau-targeting PROTAC compounds were designed and synthesized. Of these, **C8** displayed excellent degradation efficacy against p-tau, as confirmed by Western blotting in both HEK293-htau cells and htau-overexpressed mice. Additionally, **C8** was observed to show particular promise for degrading tau proteins via the ubiquitin–proteasome system in a time-dependent manner. Further research into **C8** also revealed its excellent BBB permeability and its ability to ameliorate cognitive function.

Taken together, our results indicate that we have successfully discovered a new small-molecule tau-targeting PROTAC, **C8**, which can efficiently clear p-tau and improve cognitive function. **C8** therefore represents a promising drug candidate for AD and related neurodegenerative diseases through small-molecule-mediated protein degradation technology.

## Data Availability

The original contributions presented in the study are included in the article/Supplementary Material, further inquiries can be directed to the corresponding authors.
