# Supplementary material for: Selective degradation of hyperphosphorylated tau by proteolysis-targeting chimeras ameliorates cognitive function in Alzheimer’s disease model mice
Source: Front Pharmacol. 2024 Jun 11;15:1351792. doi: 10.3389/fphar.2024.1351792 (PMC11196765; doi:10.3389/fphar.2024.1351792)
Supplement: Supplementary file 1 [file DataSheet1.ZIP › Raw data/Raw data.docx]

Fig 2A

kDa


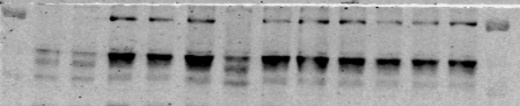


p396

-55


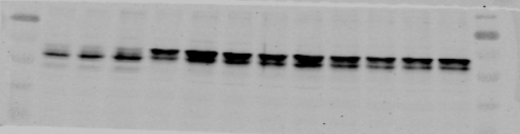


-55

p404


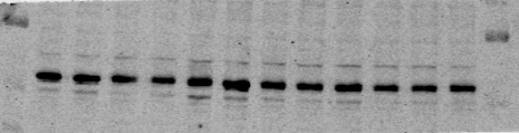


tau1

-55


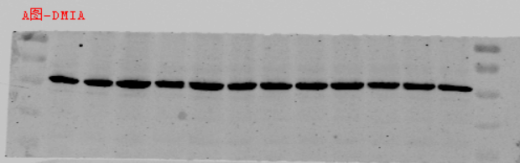


DM1A


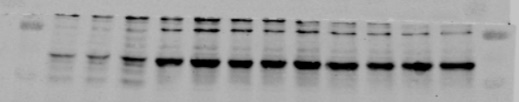


tau5

-55

-55

Fig 2B

p396


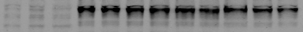


-55


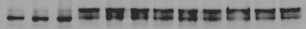


p404

-55


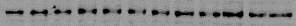


-55

tau1


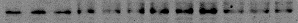


-55

tau5


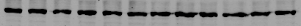


DM1A

-55

Fig 2E


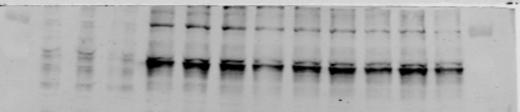


p396

-55


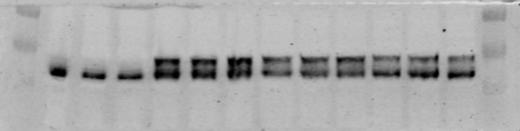


p404

-55


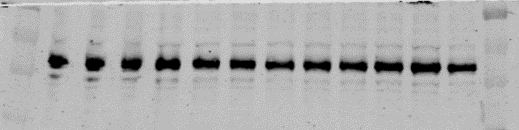


tau1

-55


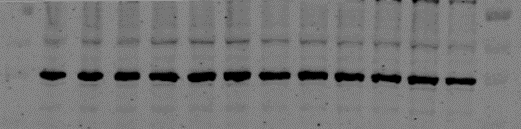

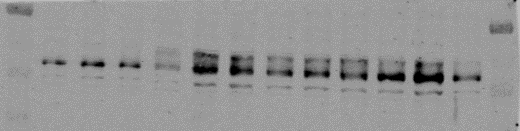


tau5

-55

-55

DM1A

Fig 3


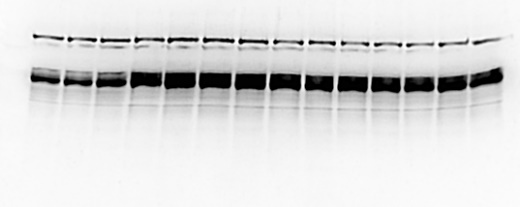

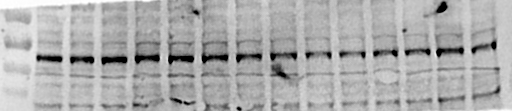

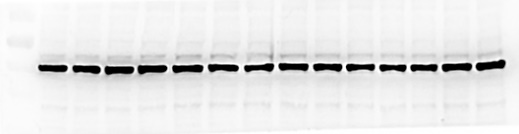

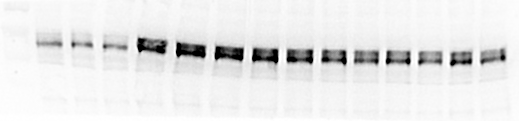

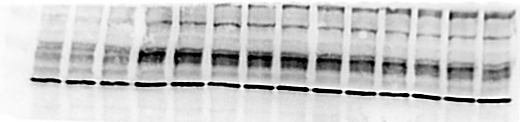

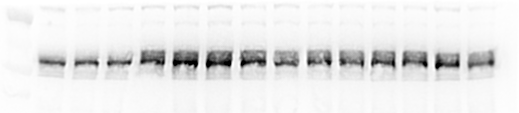


T22

tau5

DM1A

p262

p404

p396

-55

-55

-55

-100kd

-55

-55

-55

Fig 4A


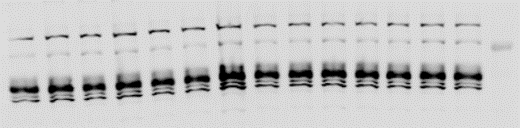


p262

-55


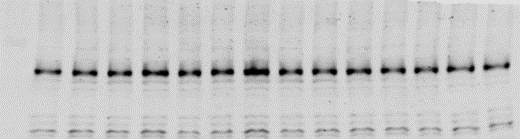

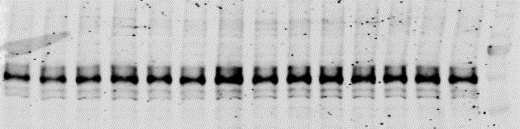

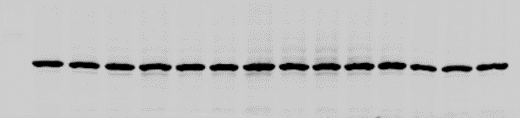

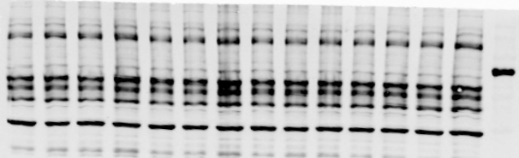


DM1A

p396

-55

p404

-55

tau5

-55

-55


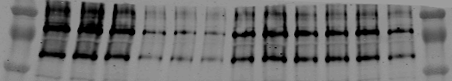
Fig 4B

-55

p262


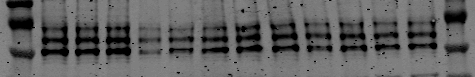


-55


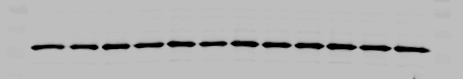

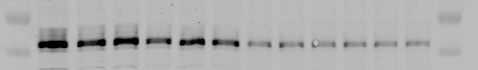

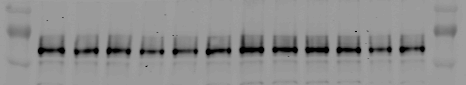


-55

-55

p396

p404

tau5

-55

DM1A

Fig 5A


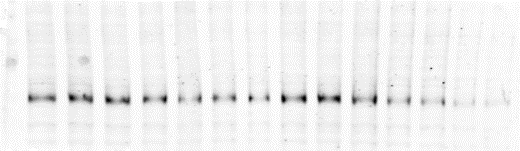

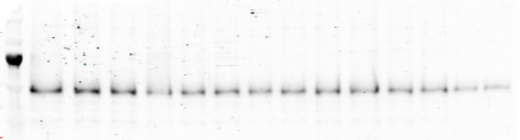

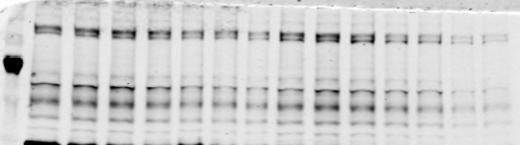

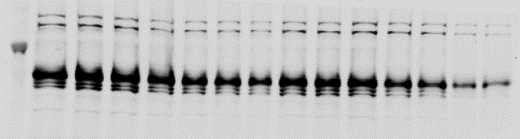


tau5

p404

p396

p262

-55

-55

-55

-55


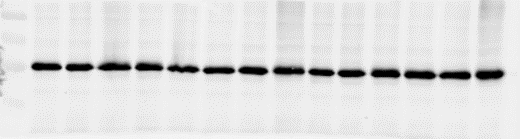


-55

DM1A

Fig 5B


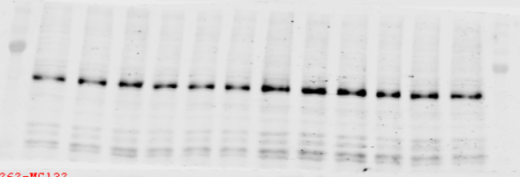

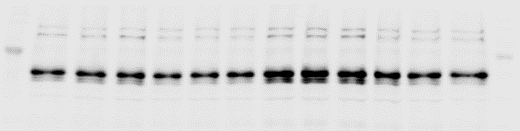

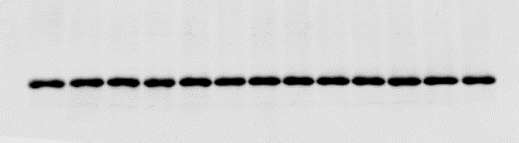

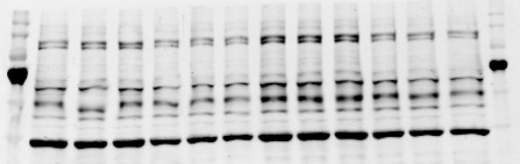

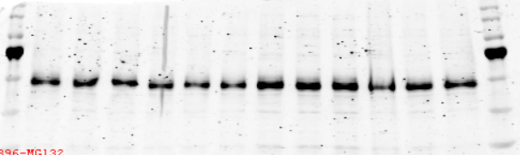


tau5

DM1A

p262

-55

p396

-55

p404

-55

-55

-55

Fig 8A


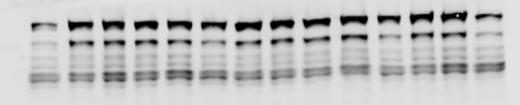


p262

-100kd

-55


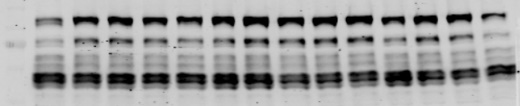


-55

-100

p404


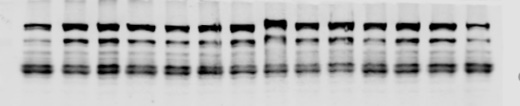


-55

-100

tau5


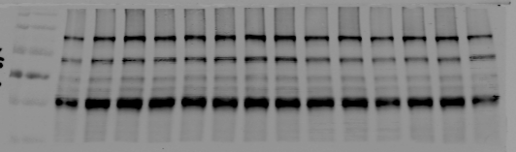


-55

-100

p396


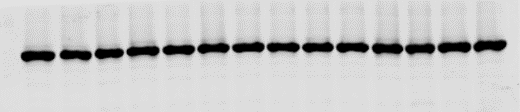


-55

DM1A

Fig 8D

p262


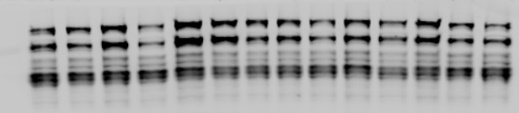


-55

-100


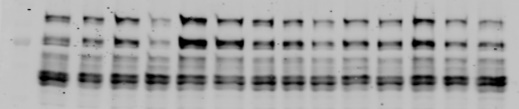


-55

-100

p404


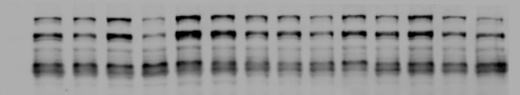


-55

-100

tau5


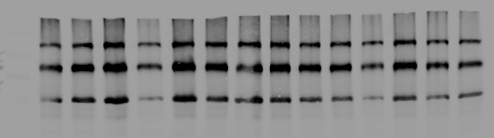


-55

-100

p396


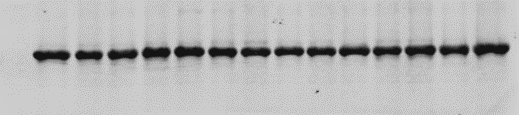


-55

DM1A

Fig 8G


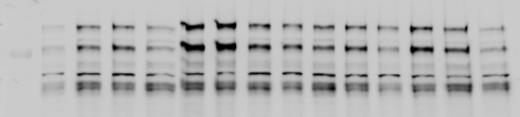


p262

-55

-100


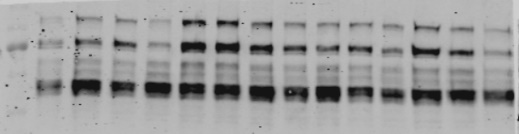


p404

-55

-100

tau5
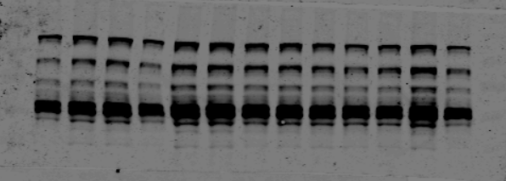


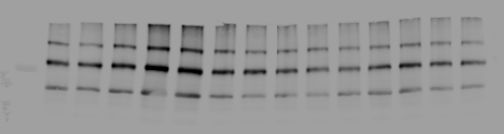


-55

-100

p396


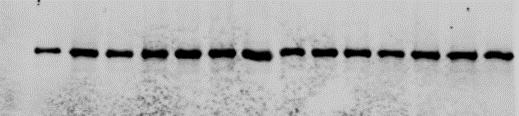


-55

DM1A

Fig. 9

tau5

tau5


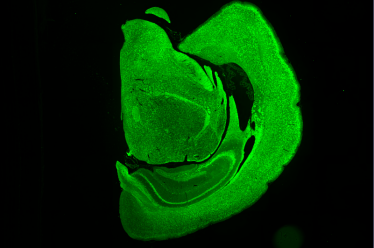

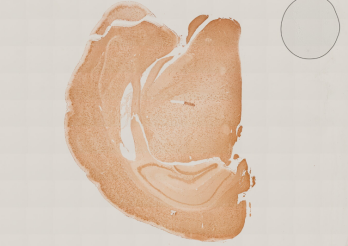


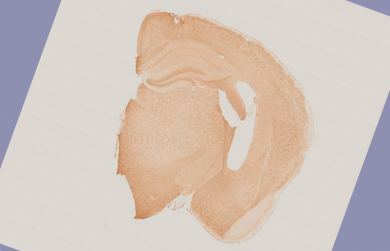


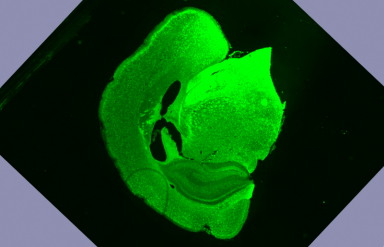


AT8


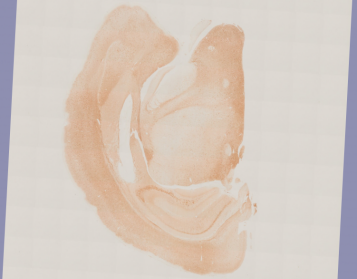

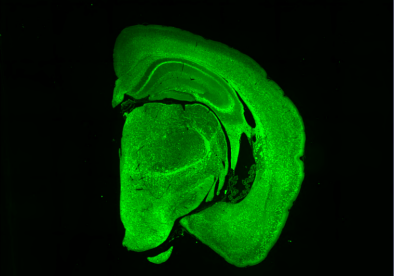


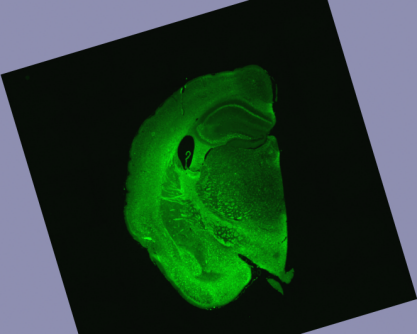

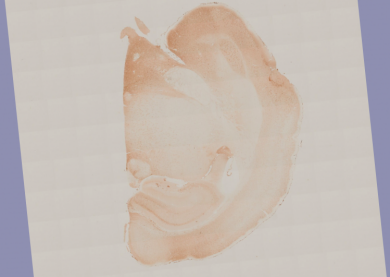


*Supplementary Material*

Supplementary Figure 18


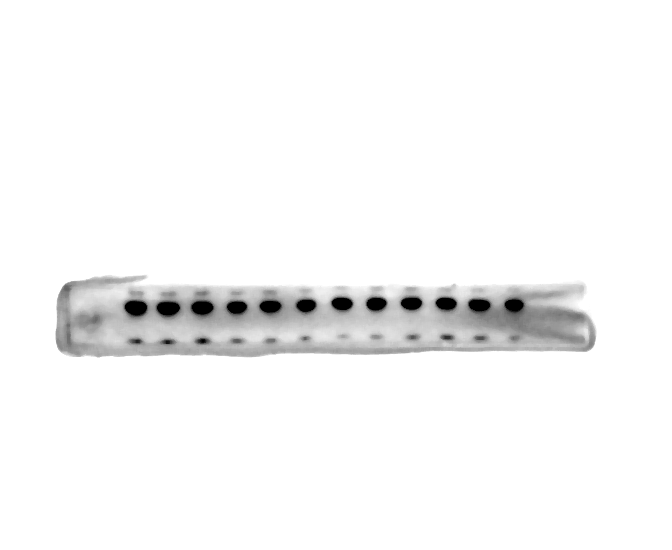

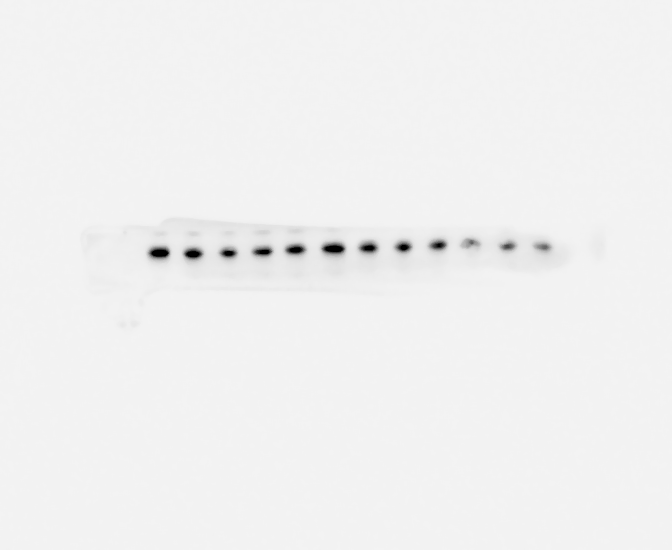

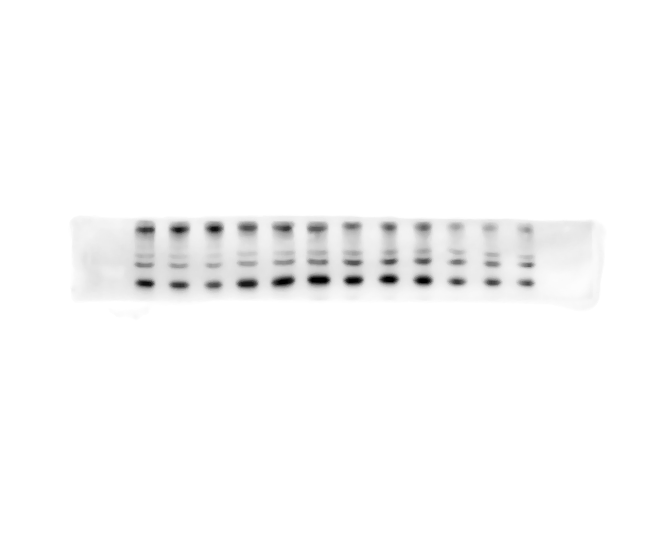

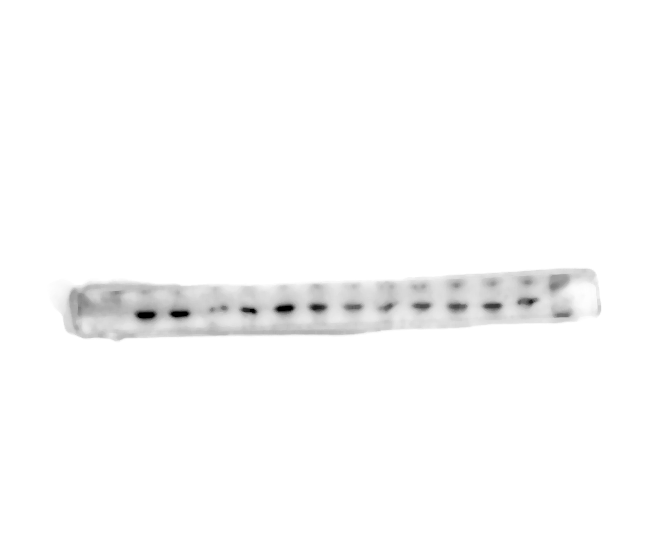
A

p404

p396

p262

-55

-55

-55

DM1A

-55

C


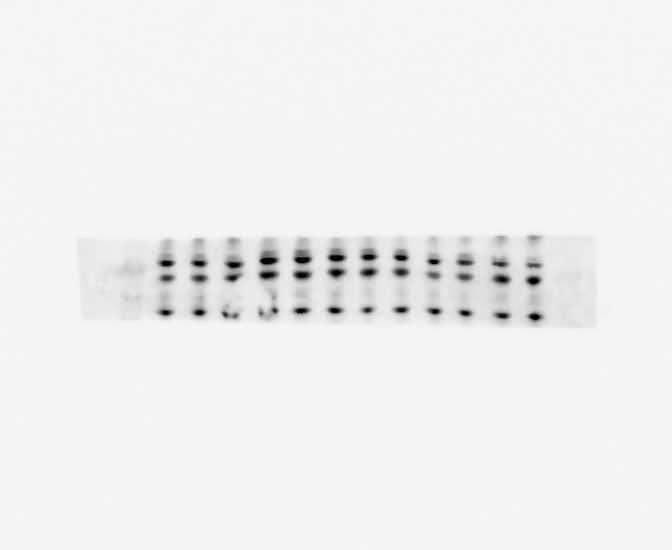


p262

-55


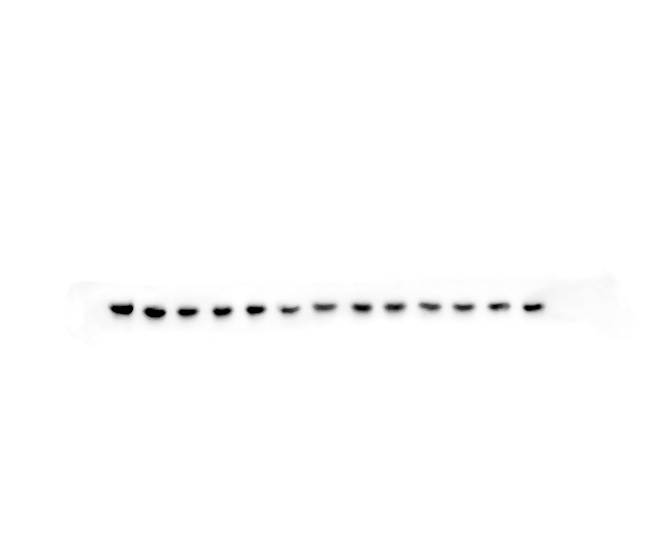

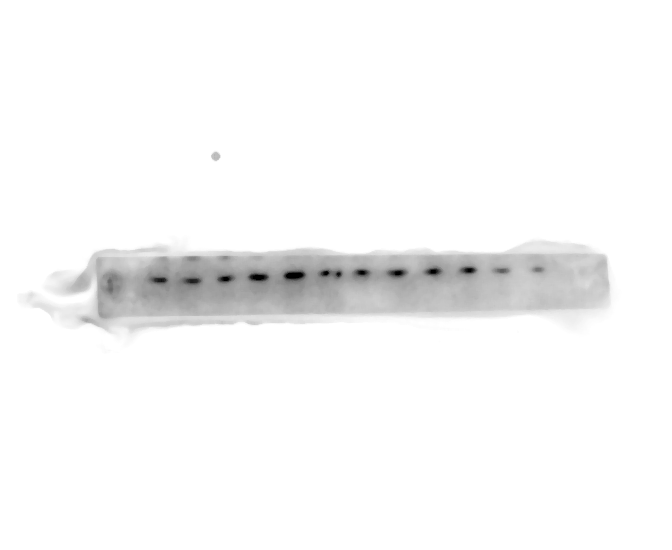

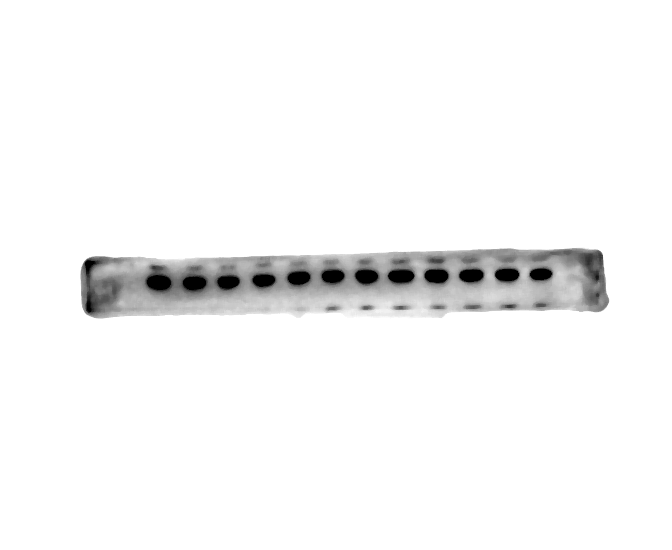


DM1A

p396

p404

-55

-55

-55
