# Supplementary material for: Selective degradation of hyperphosphorylated tau by proteolysis-targeting chimeras ameliorates cognitive function in Alzheimer’s disease model mice
Source: Front Pharmacol. 2024 Jun 11;15:1351792. doi: 10.3389/fphar.2024.1351792 (PMC11196765; doi:10.3389/fphar.2024.1351792)
Supplement: Supplementary file 2 [file Image1.pdf]

Supplementary Material

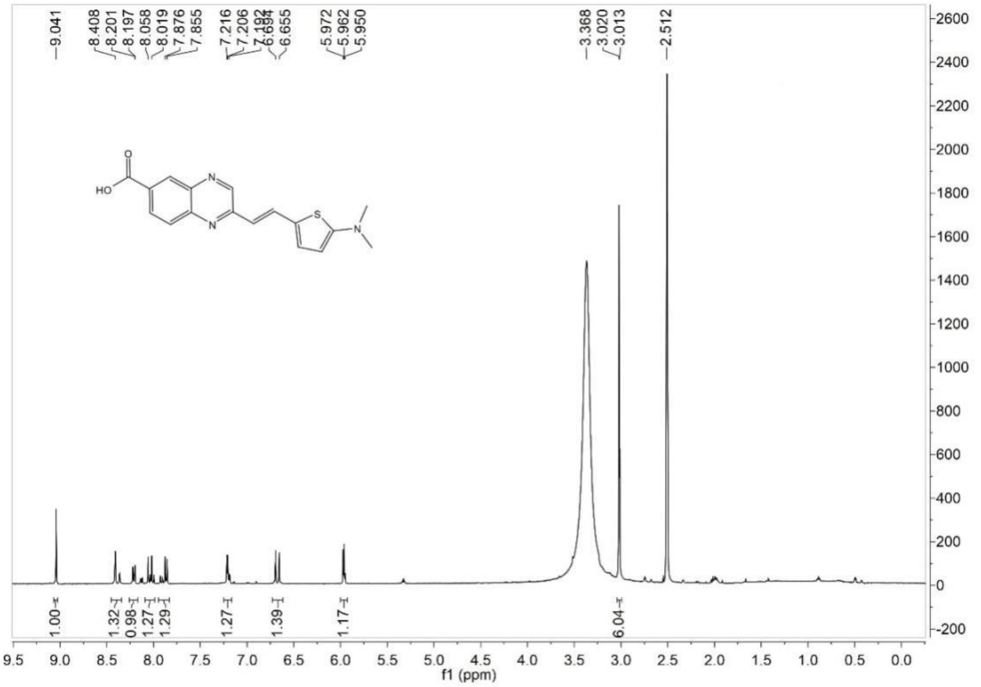

Supplementary Figure 1. ^{1}H NMR of compound 12.

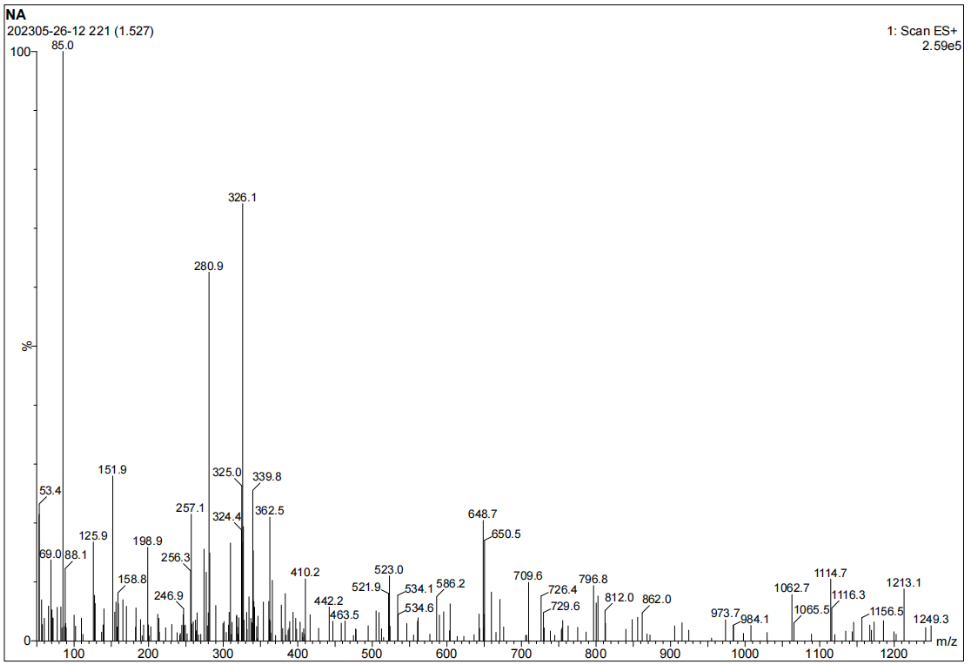

Supplementary Figure 2. MS of compound 12.

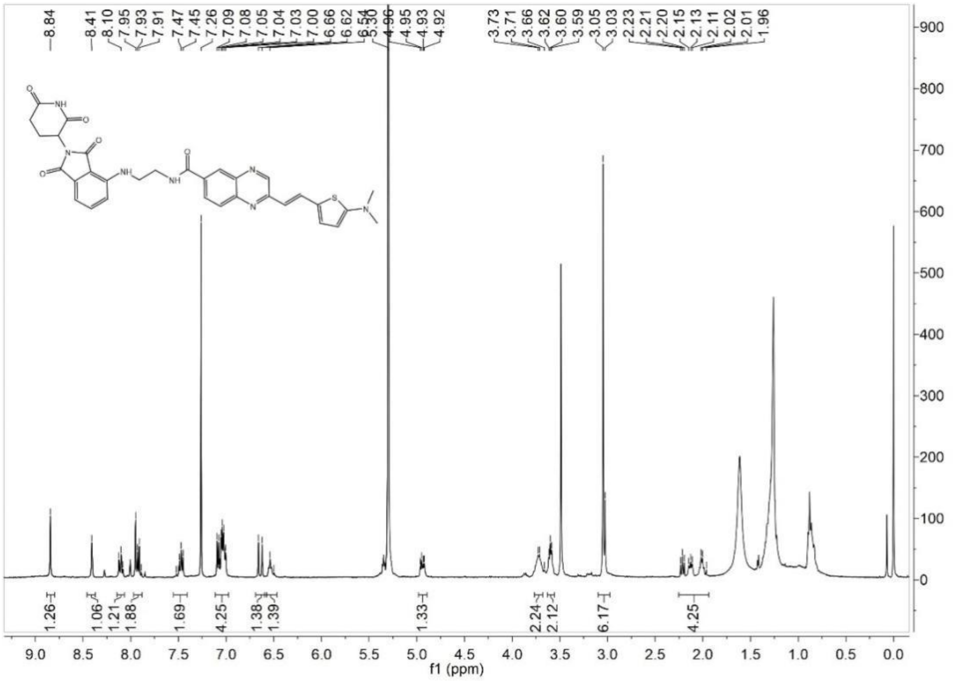

Supplementary Figure 3. ^{1}H NMR of C2.

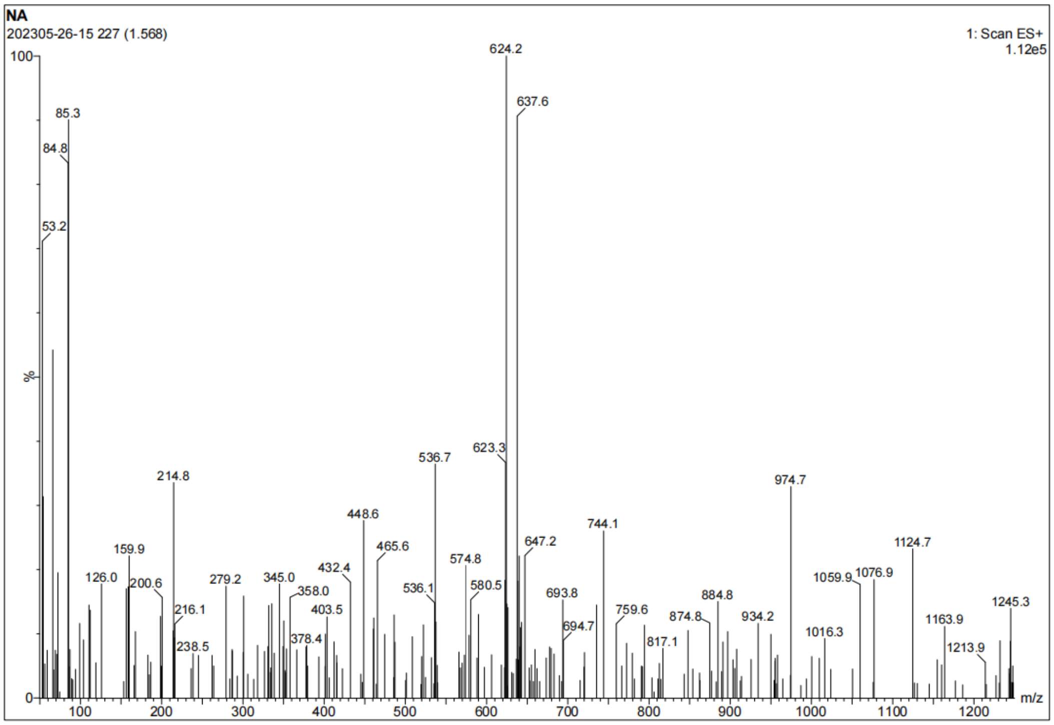

Supplementary Figure 4. MS of C2.

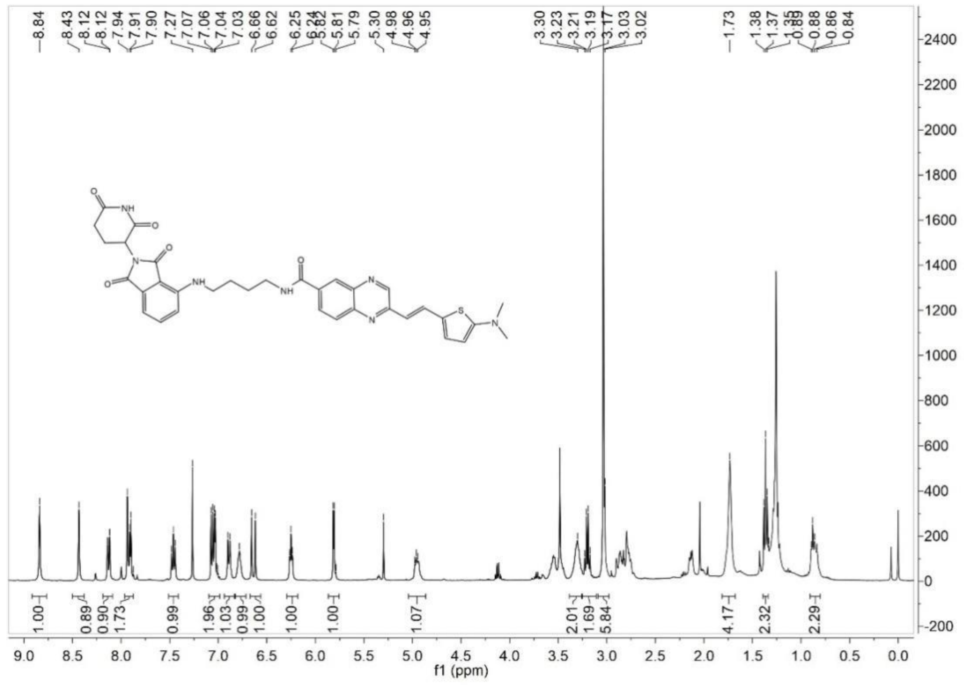

Supplementary Figure 5. ^{1}H NMR of C4.

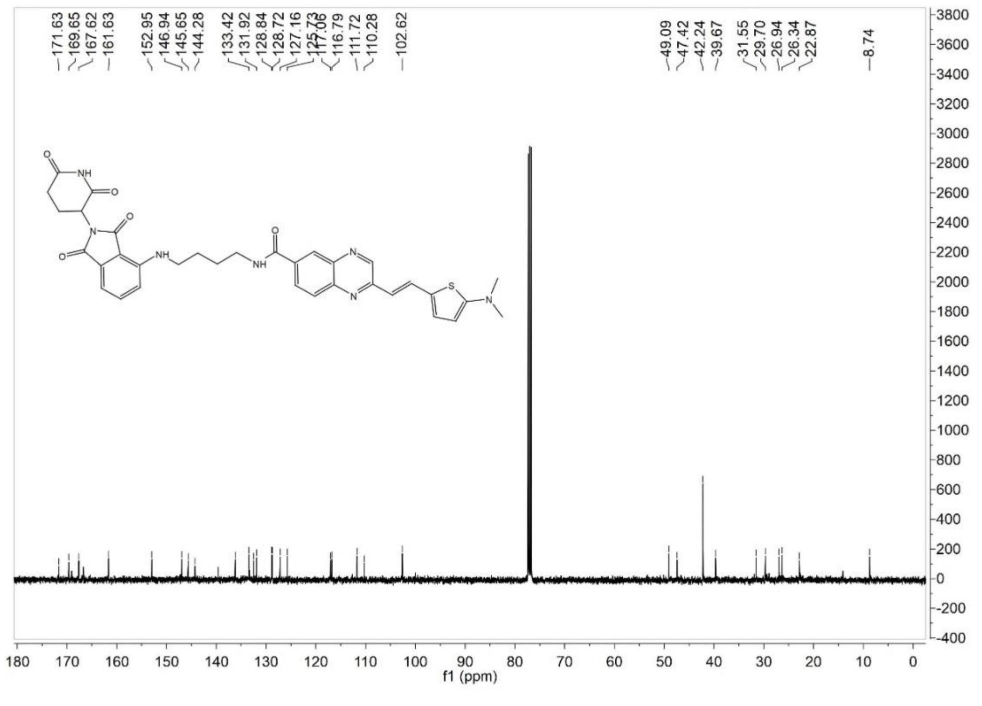

Supplementary Figure 6. ^{13}C NMR of C4.

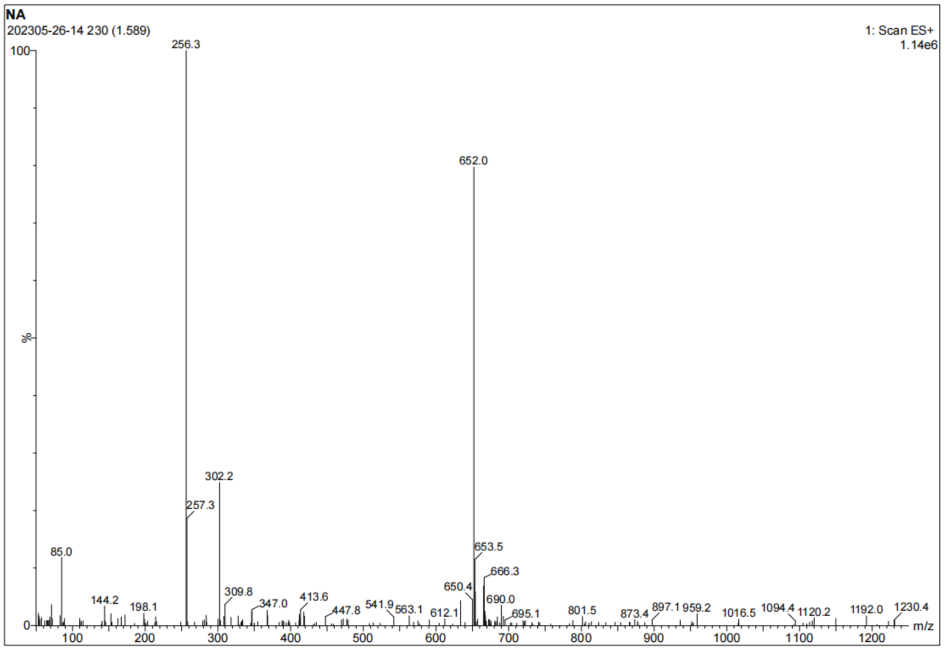

Supplementary Figure 7. MS of C4.

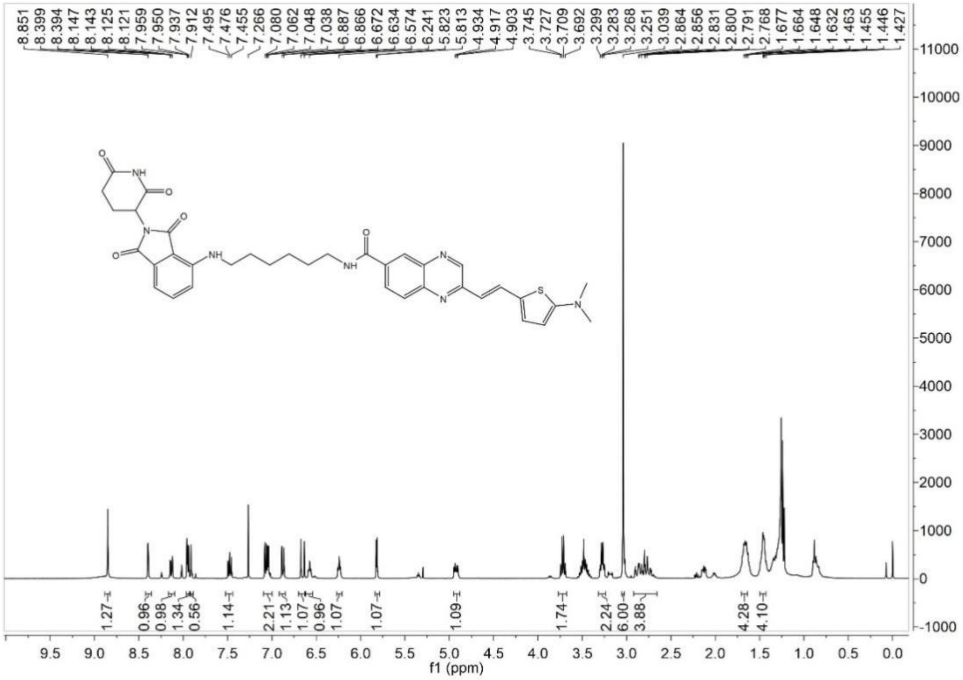

Supplementary Figure 8. ^{1}H NMR of C6.

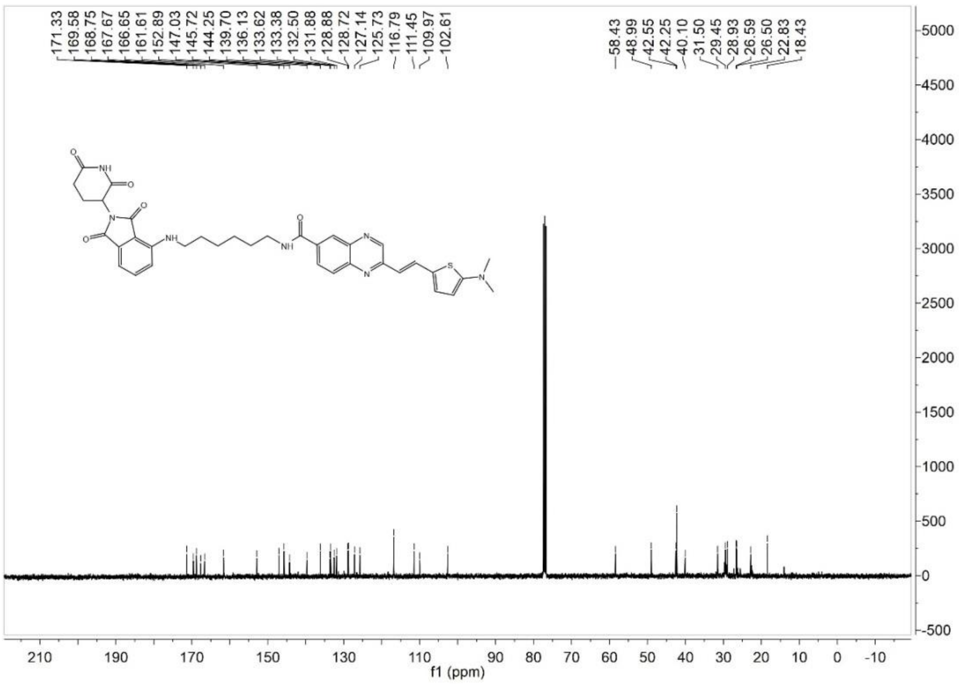

Supplementary Figure 9. ^{13}C NMR of C6.

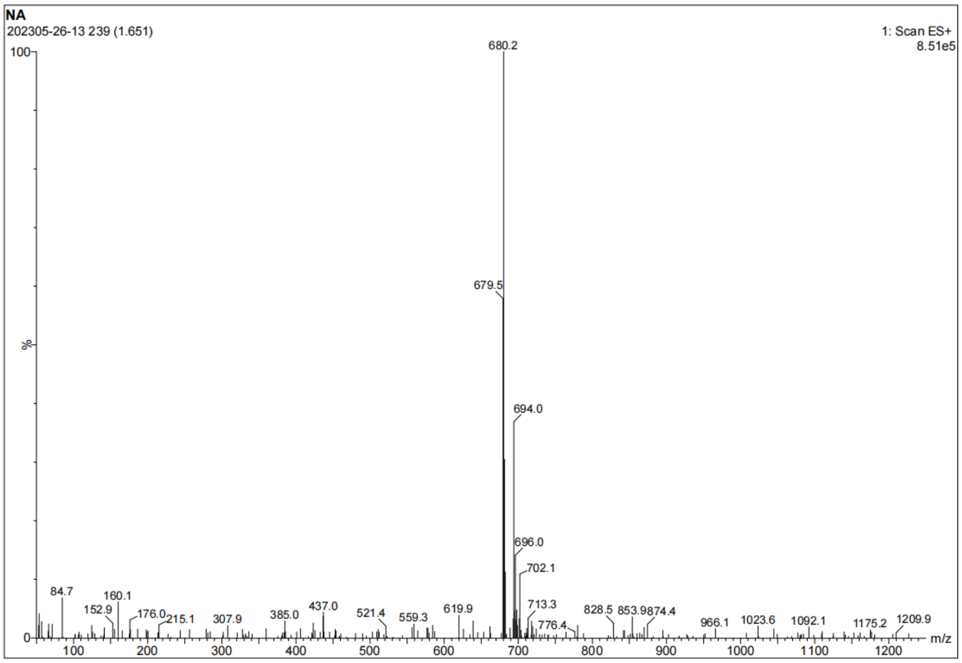

Supplementary Figure 10. MS of C6.

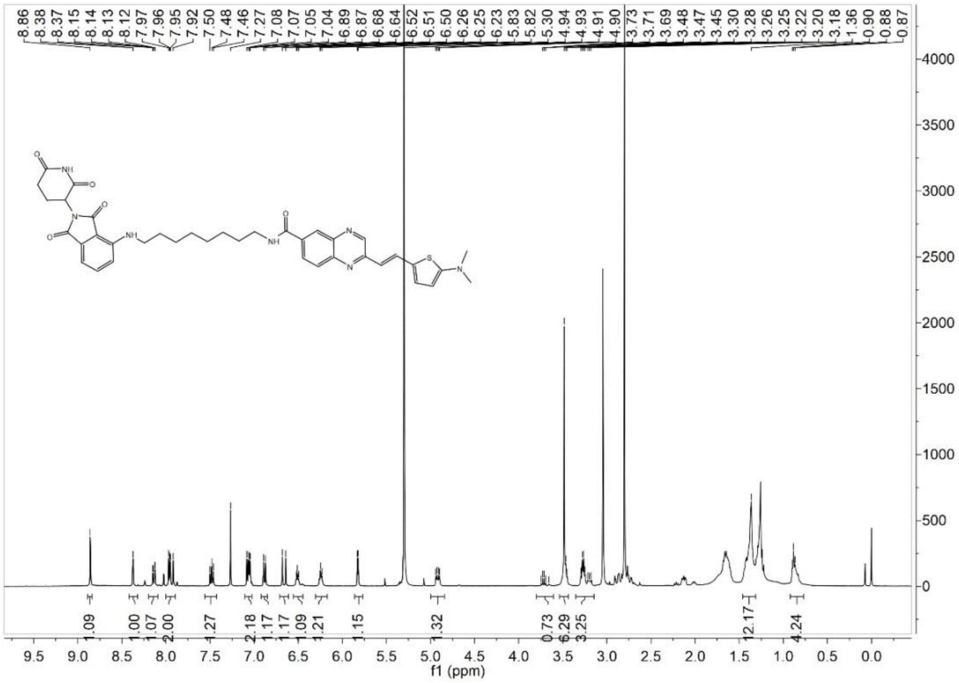

Supplementary Figure 11. ^{1}H NMR of C8.

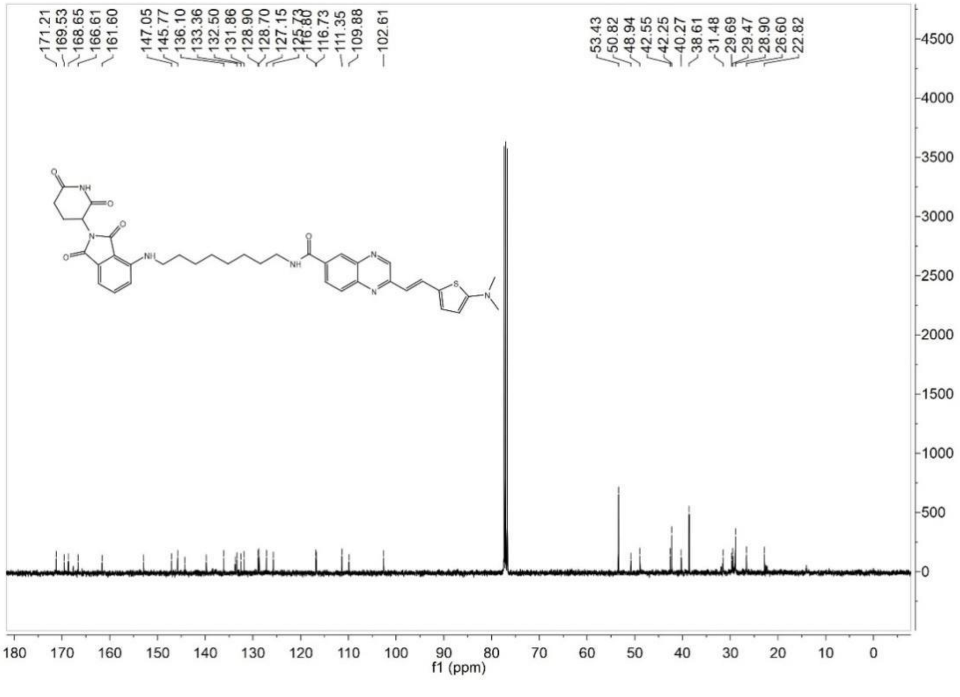

Supplementary Figure 12. ^{13}C NMR of C8.

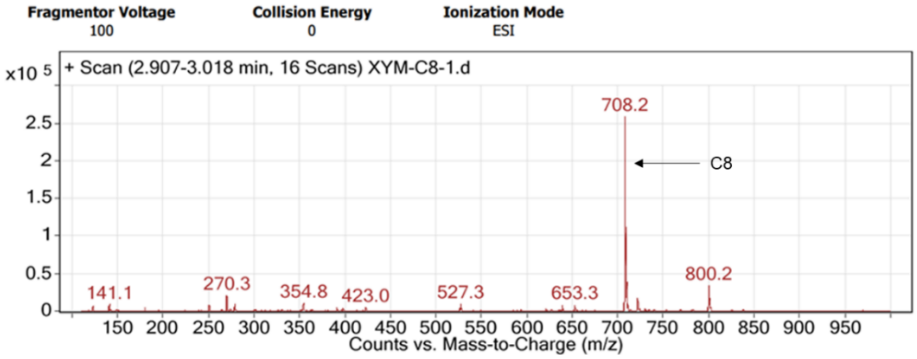

Supplementary Figure 13. MS of C8.

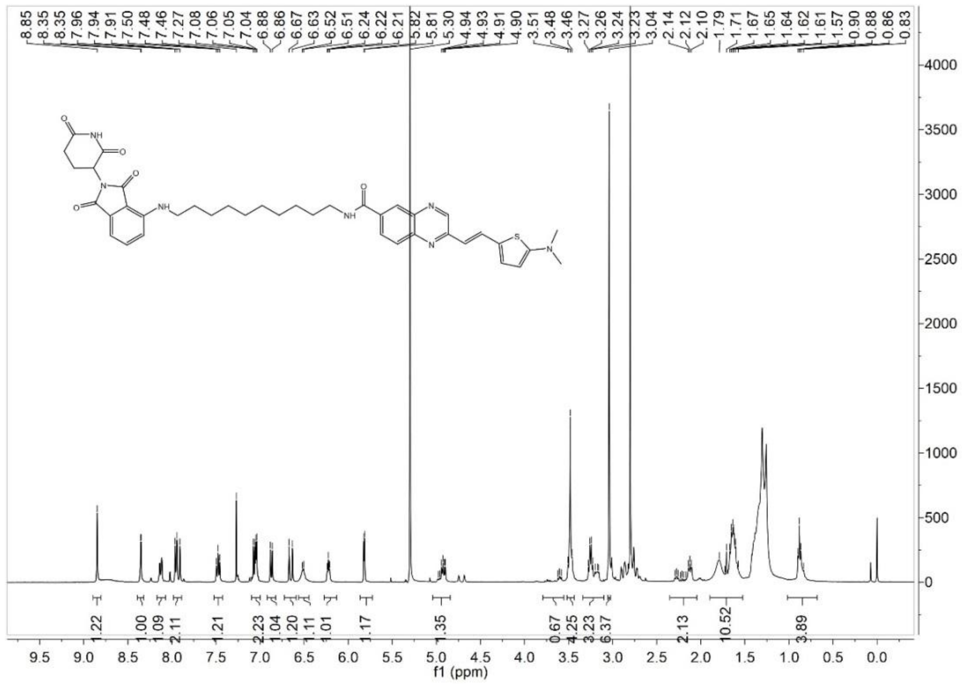

Supplementary Figure 14. ^{1}H NMR of C10.

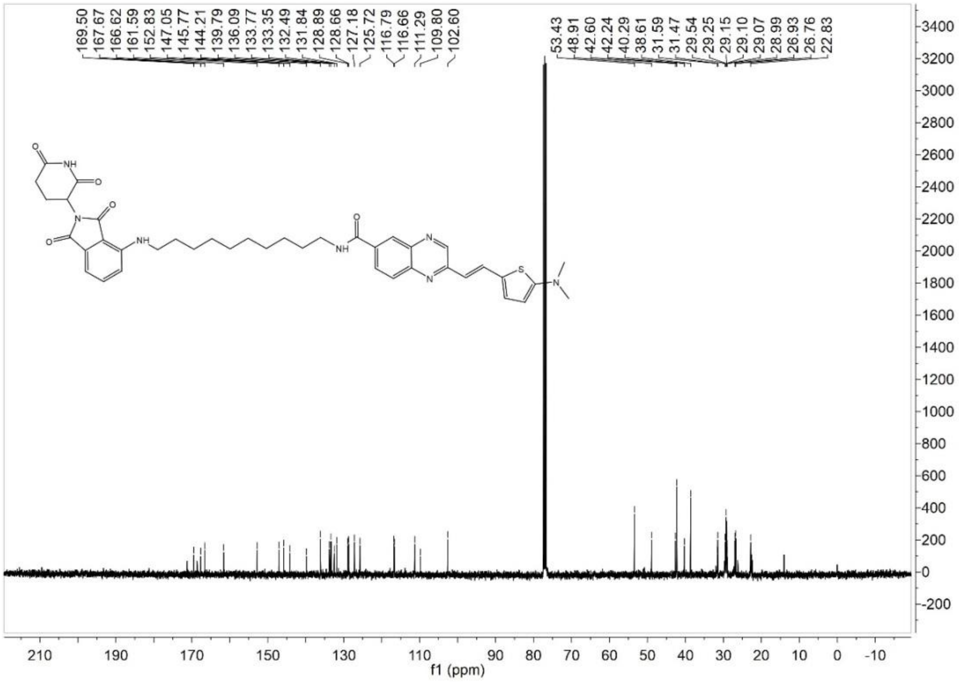

Supplementary Figure 15. ^{13}C NMR of C10.

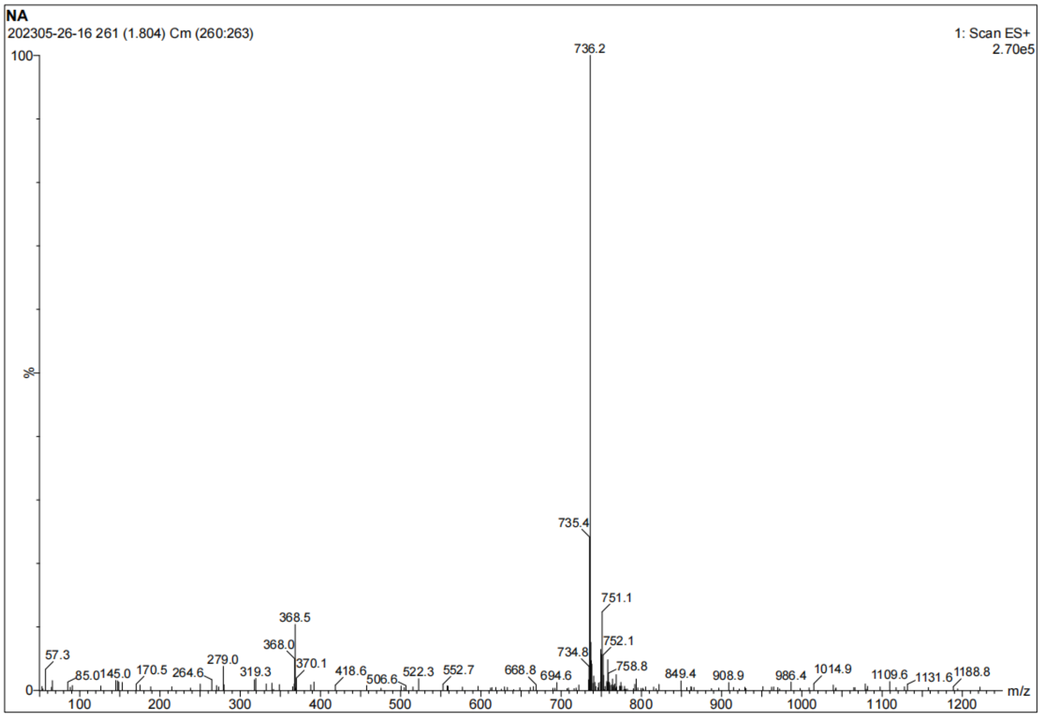

Supplementary Figure 16. MS of C10.

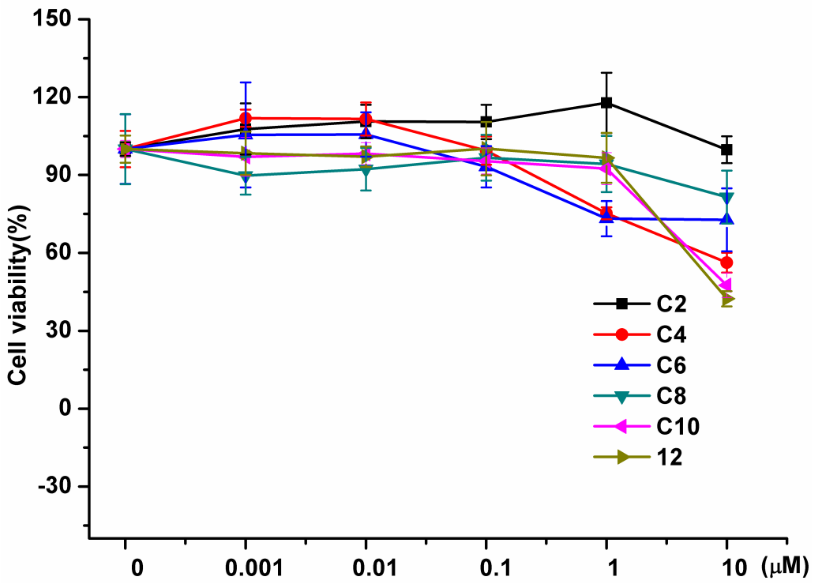

Supplementary Figure 17. Evaluation of the PROTACs cytotoxicity.

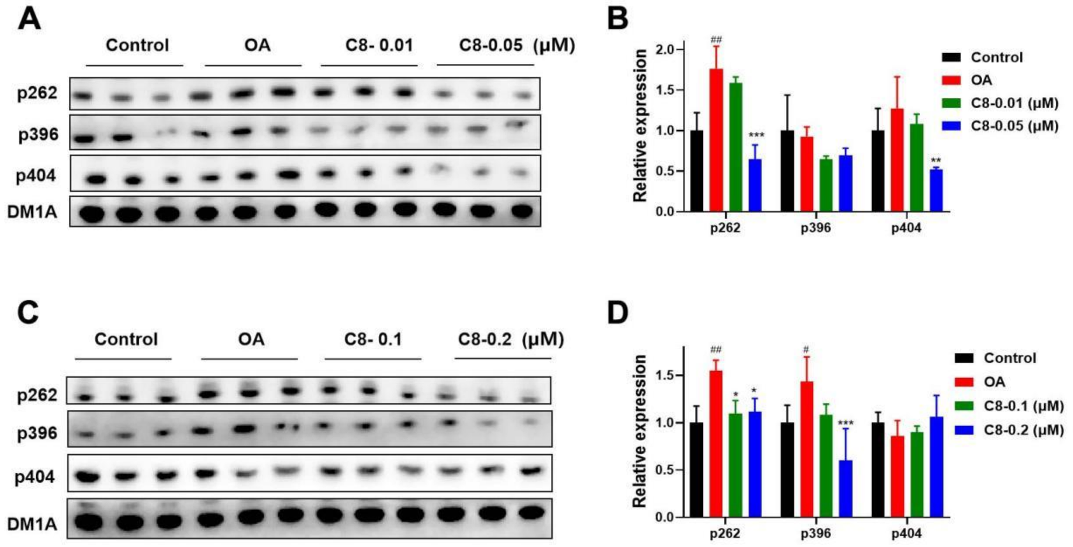

Supplementary Figure 18. C8 induced the clearance of p-tau in SH-SY5Y cells at multiple doses. C8 induced the clearance of p-tau as observed using western blotting. Data are expressed as the mean ± SEM, $^{\#}p < 0.05$, $^{\#\#}p < 0.01$, $^{\#\#\#}p < 0.001$ vs. Control. $*p < 0.05$, $**p < 0.01$, $***p < 0.001$ vs. OA. n=3.
